# Better understanding the phenotypic effects of drugs through shared targets in genetic disease networks

**DOI:** 10.3389/fphar.2024.1470931

**Published:** 2025-01-22

**Authors:** Elena Díaz-Santiago, Aurelio A. Moya-García, Jesús Pérez-García, Raquel Yahyaoui, Christine Orengo, Florencio Pazos, James R. Perkins, Juan A. G. Ranea

**Affiliations:** ^1^ Department of Molecular Biology and Biochemistry, University of Malaga, Malaga, Spain; ^2^ Laboratory of Inherited Metabolic Diseases and Newborn Screening, Malaga Regional University Hospital, Malaga, Spain; ^3^ Instituto de Investigación Biomédica de Málaga y Plataforma en Nanomedicina-IBIMA Plataforma BIONAND, Malaga, Spain; ^4^ Department of Structural and Molecular Biology, University College London, London, United Kingdom; ^5^ Computational Systems Biology Group, Systems Biology Department, National Centre for Biotechnology (CNB-CSIC), Madrid, Spain; ^6^ CIBER de Enfermedades Raras, Instituto de Salud Carlos III, Madrid, Spain; ^7^ Spanish National Bioinformatics Institute (INB/ELIXIR-ES), Instituto de Salud Carlos III (ISCIII), Madrid, Spain

**Keywords:** drug effects, side effects, adverse effects, intended effects, networks, diseases, targets, structural domains

## Abstract

**Introduction:**

Most drugs fail during development and there is a clear and unmet need for approaches to better understand mechanistically how drugs exert both their intended and adverse effects. Gaining traction in this field is the use of disease data linking genes with pathological phenotypes and combining this with drugtarget interaction data.

**Methods:**

We introduce methodology to associate drugs with effects, both intended and adverse, using a tripartite network approach that combines drug-target and target-phenotype data, in which targets can be represented as proteins and protein domains.

**Results:**

We were able to detect associations for over 140,000 ChEMBL drugs and 3,800 phenotypes, represented as Human Phenotype Ontology (HPO) terms. The overlap of these results with the SIDER databases of known drug side effects was up to 10 times higher than random, depending on the target type, disease database and score threshold used. In terms of overlap with drug-phenotype pairs extracted from the literature, the performance of our methodology was up to 17.47 times greater than random. The top results include phenotype-drug associations that represent intended effects, particularly for cancers such as chronic myelogenous leukemia, which was linked with nilotinib. They also include adverse side effects, such as blurred vision being linked with tetracaine.

**Discussion:**

This work represents an important advance in our understanding of how drugs cause intended and adverse side effects through their action on disease causing genes and has potential applications for drug development and repositioning.

## 1 Introduction

Although most pharmaceutical drugs interact with a primary protein target, they also frequently interact with off-target proteins (Moya-García et al., 2017; Chaudhari et al., 2017; Chaudhari et al., 2020; Kabir and Muth 2022). Target interaction is usually responsible for the intended effects of the drug. However, both target and off-target reactions can lead to additional consequences, like side effects and adverse effects (Lee et al., 2011; Chaudhari et al., 2017; Chaudhari et al., 2020; Kabir and Muth 2022).

These unwanted effects are a problem for drug discovery. They have led to a wide range of drugs being pulled from the market (Onakpoya et al., 2016; Bremner, 2021; Czernichow and Batty, 2010; Sharav and Benoliel, 2008; Furberg and Pitt, 2001; Li Wan Po and Zhang, 1998) and have clear implications for patient health (Sultana et al., 2013) as well as an important economic impact, given the estimated average cost to take a new drug to market is around $985 million (Wouters et al., 2020). The effect of a drug is due to the interactions it establishes with various targets in different cells and tissues throughout the body (Davis, 2020). One of the reasons drug development often fails is that these interactions are not well understood (Zhou et al., 2016). Therefore, there is an unmet need to understand the mechanisms of action of drugs in order to address the different challenges in current drug development (Chaudhari et al., 2020; Lee et al., 2011; Iwata et al., 2013).

Previous studies have tried to repurpose drugs for new diseases based on known and predicted targets (Jarada et al., 2020; Kinnings et al., 2009; Yang et al., 2014; Sirota et al., 2011; Joshua Swamidass, 2011), as well as methods employing expression profile similarity (Jarada et al., 2020; Huang et al., 2015; Rukov et al., 2014; Hu and Agarwal, 2009; Lamb et al., 2006). Some studies have looked at repurposing drugs based on similarities in terms of their side-effect profiles (Zeng et al., 2019; Ye et al., 2014; Wang et al., 2013; Yang and Agarwal, 2011; Campillos et al., 2008; Plenge, 2016). However, only a few studies have combined data related to drug-target interactions with data on how these targets can lead to pathological phenotypes as a way to study their adverse effects, based on the central tenet that the pathological phenotypes associated with variants in a given protein can also occur when that protein is drugged. One such study showed that drugs are more likely to lead to side effects in a given organ system if that drug’s protein target has been associated with a phenotype related to the same organ system (Nguyen et al., 2019). This was achieved by combining drug-target and gene-phenotype data from multiple sources, linked via the Unified Medical Language System (UMLS) (Bodenreider, 2004). In another study, Estrada et al. proposed an approach to identify drug targets based on the identification of genes with both gain-of-function (GF) and loss-of-function (LOF) mutations associated with opposite effects on the phenotype (selected targets with bidirectional effect) (Estrada et al., 2021). Another approach exploits interactions between molecules to develop a graph-based model for predicting side effects (Huang et al., 2023).

Most previous studies into drug effects have tended to focus on protein targets. However, there has been a strong push in recent years to consider the target in terms of constituent functionally, structurally and evolutionary independent units: protein domains (Wang et al., 2012; Iwata et al., 2013; Moya-García et al., 2017), allowing a more fine-grained mapping between drugs and their targets (Kruger et al., 2012). In a recent study, Moya-García et al. (2017) showed that CATH-Functional Families (FunFams) (Das et al., 2021) that were overrepresented in druggable proteins tended to have conserved drug-binding sites. Other domain-based work has sought to infer domain-side effect interactions through a learning-based approach based on a known set of drug-domain interactions (Iwata et al., 2013). Moya-García and Ranea modelled drug-domain networks to explore the role of protein domains as drug targets (Moya-García and Ranea, 2013).

In this work, we combine information on drug-target interactions and associations between phenotypes and proteins/domains. This approach enables the generation of phenotype-drug associations, providing a more comprehensive understanding of drug effects, both intended and adverse. Moreover, it facilitates insight into the molecular mechanisms that mediate these effects. We aim to tackle the main challenge of linking drugs to their effects—both desired and unintended side effects—which is a key issue in drug development. We also seek to understand the molecular details of how drugs interact with their targets, leading to these effects.

## 2 Materials and methods

The approach developed in this work, termed *Drugeff-analyser*, associates drugs with potential effects, described in terms of pathological phenotypes in the Human Phenotype Ontology (HPO) (Köhler et al., 2021). This is performed by constructing and analysing networks connecting drugs, targets and phenotypes. The results are validated using known drug-effects data, as well as data inferred from co-occurrence in the scientific literature.

**FIGURE 1:**
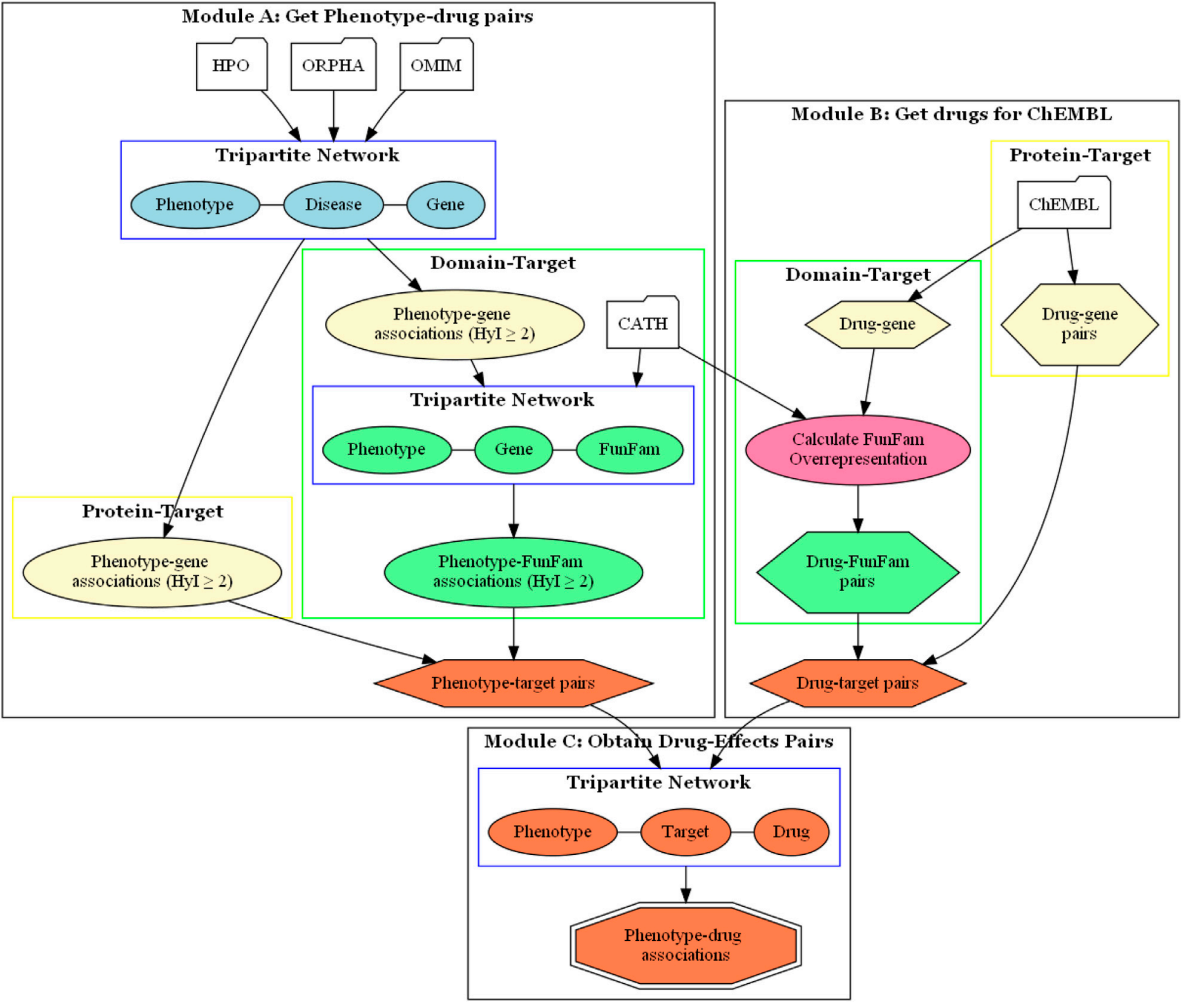
Drugeff-analyser workflow. Module **(A)** Information from OMIM or Orphanet was used to build a network to obtain phenotype-target pairs (orange hexagon). For the protein-target approach, these were taken directly from phenotype-target associations (lemon oval). For the domain-target approach these pairs were then combined with FunFam-gene relationships from the CATH database to obtain phenotype-FunFam associations (green box). There were two tripartite networks, as shown in blue boxes, both used to calculate associations. Module **(B)** The ChEMBL database was queried to obtain drug-target pairs. For the protein-target approach, drug-gene pairs (lemon hexagon) were used directly. For the domain-target approach, FunFams that were overrepresented among the drug targets were obtained (green box). **(C)** with the pairs obtained in previous modules, a tripartite network was built to associate phenotypes and drugs based on target overlap, resulting in phenotype-drug pairs (orange octagon). HyI: hypergeometric index.

We used two types of targets, proteins and protein domains. When we refer to using the protein as a target, we are talking about the complete protein. However, in reality, proteins are composed of different domains, which are responsible for carrying out the biological functions of the proteins, including interactions with drugs. We used CATH FunFams as protein domains. FunFams group different domains that share similar structures and functions, and have been used for predicting functional sites, making them suitable for studying the effects of drugs on proteins, and how this impacts on the phenotype (Dawson et al., 2017; Sillitoe et al., 2020). Thus, two methods were implemented, using the Autoflow workflow manager (Seoane et al., 2016), to associate drugs with phenotypes, based on protein-targets and protein domain-targets, respectively (Figure 1).

The methods consist of two initial steps which result in phenotype-target (Figure 1 Module A) and drug-target (Figure 1 Module B) pairs. These pairs are then combined to build a tripartite network, which is analysed using *NetAnalyzer* (Rojano et al., 2017) to produce a list of phenotype-drug pairs (Figure 1 Module C). By analyzing shared connections between the layers, one can infer relationships between the nodes, in this case between drugs and phenotypes, based on shared targets.

### 2.1 Obtaining phenotype-target pairs

Both the protein and domain-target methods start by connecting phenotypes and genes via shared diseases, according to either the OMIM or Orphanet databases, following the methods described in (Díaz-Santiago et al., 2021). Note that OMIM and Orphanet were used independently to build separate networks and analysed independently, as they gather information from different origins and with different goals (Díaz-Santiago et al., 2021). Thus, genes and phenotypes that are linked by common diseases can be deemed associated and considered phenotype-gene pairs. The significance of the association is quantified using the *NetAnalyzer* software, implementing the hypergeometric index, which has been shown to outperform other metrics when connecting phenotypes with genes and disease (Díaz-Santiago et al., 2021; Díaz-Santiago et al., 2020; Bueno et al., 2018; Rojano et al., 2017).

The Hypergeometric Index is a statistical measure used to evaluate the significance of the overlap between two sets, A and B , and is based on the hypergeometric distribution, which calculates the probability of observing a given overlap by chance. It represents the log-transformed probability that the overlap between A and B is equal or greater than what is observed. More formally, to assess the overlap between two groups A and B using the hypergeometric distribution, you typically calculate the probability of observing at least k shared elements between them, assuming random sampling from a larger population N . The equation for this hypergeometric probability is:

P(X≥k)=∑i=kmin(|A|,|B|)|A|iN−|A||B|−iN|B|

Where N : Total number of elements in the population, |A| : Size of group A , |B| : Size of group B , k : Number of shared elements between A and B , and nr the binomial coefficient, representing the number of ways to choose r items from n . Essentially, it quantifies how likely it is that the number of shared interactions between the two sets could happen randomly. A lower probability (and so higher Hypergeometric Index value) indicates that the overlap is highly unlikely to be due to chance, suggesting a meaningful or significant interaction between A and B . We applied various thresholds, between 2 and 3.5, which correspond to *p*-values of 0.01 to 0.0003, to filter weak interactions (Bass et al., 2013).

By using a hypergeometric index threshold, we aim to filter out unspecific phenotypes that can appear in a large number of diseases. These phenotype-gene pairs are used directly as the phenotype-target pairs for the protein-target based method, as shown in Figure 1 Module A, in the sub-workflow on the left.

For the domain-target based method, we start with the phenotype-target associations. Each gene is then paired with any CATH FunFam (Sillitoe et al., 2021) that contains domains belonging to the protein product of the gene (Figure 1 Module A, sub-workflow on the right). FunFam functional domain data was obtained from the CATH database (release v4.3.0). Each gene in the set of phenotype-target pairs can contain one or more FunFams, and domains from the same FunFam can be found in multiple proteins. Therefore, we analyze the significance of the associations between phenotypes and FunFams using the hypergeometric index implemented in *NetAnalyzer*, using a threshold of 2, in the same way as described above to associate phenotypes with genes.

### 2.2 Obtaining drug-target pairs

Drug-protein target pairs were obtained from the ChEMBL database (version 29 Davies et al., 2015; Mendez et al., 2019), using the following criteria based on a previous study (Moya-García et al., 2017):

• Small molecule with therapeutic application (Therapeutic Flag = 1).
• Direct binding interaction with single protein (Assay Type = B; Relationship Type = D; Target Type = Single Protein).
• Filtering out weak activities (pchembl value ≥ 6).
• Drugs in every stage of development were considered.

The drug-protein pairs were used to associate drugs with phenotypes in the protein-target method (Figure 1 Module B, sub-workflow on the right). For the domain-target method, the drug-protein pairs were further decomposed into drug-FunFam pairs, where FunFams represent functional domain families (Figure 1 Module B, sub-workflow on the left). Given that each protein in the drug-protein pairs list can contain one or more FunFams, and domains from the same FunFam can be found in multiple proteins, we evaluated whether each FunFam is significantly overrepresented among the targets of a given drug using the expected probability and binomial test. As such, we could associate the drugs with the FunFams significantly overrepresented among their respective targets, obtaining a set of drug-FunFam pairs using a *p*-value threshold of 0.05. Full details are given in (Moya-García et al., 2017).

### 2.3 Combining pairs and associating drugs with intended and adverse effects via tripartite network analysis

The phenotype-target and drug-target pairs are then combined to produce phenotype-target-drug tripartite networks (Figure 1 Module C). This is performed separately for the protein and domain-target workflows. Phenotypes were associated with drugs based on overlap across shared targets, again using the hypergeometric index. Different thresholds of this index were used in order to assess the effect on the numbers of phenotype-drug associations found and the performance of the method. We used all drugs obtained from ChEMBL connected to at least one target in the drug-target pairs, and all phenotypes connected to at least one target in the phenotype-target pairs.

### 2.4 Assessing the overlap between the phenotype-drug associations obtained and known drug-effects

To assess the overlap between the phenotype-drug associations obtained with our methodology and known phenotype-drug associations, two different analyses have been conducted. The first one assesses the overlap of the obtained phenotype-drug pairs with SIDER (Kuhn et al., 2016), a gold standard database of drug effects. The second analysis examined the overlap between the obtained phenotype-drug associations with those associations derived from co-occurrence in the scientific literature.

Our two workflows associate HPO phenotypes with drugs in order to better understand potential phenotypes that might result from the intake of these drugs, under the assumption that these phenotypes represent potential adverse/side effects, as well as phenotypes that the drug is intended to combat.

For the SIDER comparison, a list of drug-effects was taken from the SIDER database (version 4.1 Kuhn et al., 2016). SIDER is considered a gold-standard database of drugs effects; however, it does not annotate its drugs with ChEMBL IDs, nor does it contain effects in terms of pathological phenotype HPO terms; rather it uses UMLS terms. Therefore, in order to compare our phenotype-drug pairs (i.e., HPO-ChEMBL) with SIDER, we had to perform the following steps: Firstly, the *Biothings Client* (v0.2.6 https://pypi.org/project/biothings-client) was used to query SIDER directly through the MyChem.Info database, which allowed us to connect ChEMBL IDs directly with their associated effects in the SIDER database (Kuhn et al., 2016). Secondly, the UMLS terms were mapped to HPO phenotypes using the OXO ontology mapping tool, via the *OxO REST API* (https://www.ebi.ac.uk/spot/oxo/). These steps led to the construction of a SIDER-derived list of gold standard phenotype (effect)-drug pairs that could be compared directly to our phenotype-drug associations.

An important caveat must be considered when using SIDER to assess our data: its contents are limited only to marketed drugs. As such, only a small percentage of drugs from our phenotype-drug lists, which are constructed using drugs from ChEMBL database in all phases of development, are included in SIDER. Therefore, when comparing overlap between our lists and the gold-standard list, we initially refined our dataset by considering all pairs that comprised drugs and phenotypes that appeared at least once in SIDER.

To evaluate the significance of the overlap between our phenotype-drug associations and SIDER, we generated randomized lists of phenotype-drug association, following the links-based randomization method described in previous work (Díaz-Santiago et al., 2020). The phenotypes and drugs were kept the same, but the connections between them were randomized. Randomization was performed in this way to ensure that the prevalence of phenotypes and drugs remained the same. We estimated the performance of our methods by repeating this 100 times and comparing the overlap of these randomized lists with SIDER by calculating the ratio of real vs. random, where real refers to the number of associated phenotype-drug pairs in our list that are also found in SIDER and random refers to the average overlap between the randomized pairs list and SIDER.

As a second validation we also looked at overlap between our lists of phenotype-drug associations and lists of phenotype-drug associations based on co-occurrence in the scientific literature. To achieve this, we used the methodology described previously (Pazos et al., 2022). In brief, we obtained the list of PubMed entries mentioning a specific drug by querying the *NCBI Entrez API* for articles that include the name of each drug or any of its ChEMBL synonyms in any field. The same was done for each HPO term name and its synonyms. Then, the set of PubMed entries mentioning an HPO term together with a given drug is inferred as the intersection of the sets mentioning each individually. Taking into account the number of articles mentioning the drug, the number mentioning the HPO and the number mentioning both, as well as the whole size of PubMed, a statistical test is applied to assess the significance of each HPO-drug pair in terms of co-occurrence (Pazos et al., 2022). Co-occurring HPO-drug pairs with a *p*-value < 0.001 were considered significant.

Once this list of HPO-drug pairs co-occurring in the literature was obtained, we calculated the overlap between these pairs and our lists of phenotype-drug associations, generated using our analysis workflow. Overlap was also calculated using randomized phenotype-drug pairs, obtained by randomizing the connections between the pairs in the lists generated by our analysis workflow, performing each randomization procedure 100 times. The ratio between real and random was then calculated, in the same manner as for the SIDER data described above.

## 3 Results

We used two workflows to associate drugs with pathological phenotypes. One used shared proteins as a way to link these entities, while the other used protein domains (Figure 1).

### 3.1 Phenotype-target pairs

The first step in these workflows was to obtain phenotype-target pairs. For the protein-target workflow, the total numbers of pairs and phenotypes found for different hypergeometric index thresholds are shown in Table 1 (Phenotype-Gene Pairs).

**TABLE 1:** Total numbers of phenotype-gene and phenotype-FunFam pairs obtained at different hypergeometric index thresholds using the protein-target workflow and domain-target workflow.

	Phenotype-gene pairs					
	OMIM			Orphanet		
	Pairs	Phenotypes	Genes	Pairs	Phenotypes	Genes
Total	103,030	7,304	4,485	134,021	6,670	3,173
HyI ≥ 2	41,224	7,279	4,351	48928	6,646	3,153
HyI ≥ 3	9,674	5,662	2,967	10512	4,121	2,294
HyI ≥ 3.5	3,458	2,480	1,475	4,132	1,653	650

	Phenotype-FunFam Pairs					
	OMIM			Orphanet		
	Pairs	Phenotypes	FunFams	Pairs	Phenotypes	FunFams
Total	114,772	6,886	9,286	132,419	6,419	7,078
HyI ≥ 2	112,515	6,886	9,279	127,034	6,419	7,009
HyI ≥ 3	51,394	6,252	7,807	55,630	5,870	5,447
HyU ≥ 3.5	19,390	4,580	5,429	17,963	3,966	3,426

HyI, hypergeometric index; FunFam, CATH Functional Family.

As expected, these numbers decrease at more restrictive association thresholds. This pattern is consistent for both OMIM and Orphanet. The pairs with the highest association scores using the OMIM data are shown in Table 2. The two associations with the highest scores are between HPO term *somatic mutation* and two genes encoding proteins with clear roles in this process, *KRAS* and *PIK3CA* (Luo et al., 2020; Palomba et al., 2012). In fact, of the 13 OMIM diseases associated with *PIK3CA*, 10 hold this phenotype. Similarly, of the 12 OMIM diseases associated with *KRAS*, 9 hold this phenotype. Moreover, of the 75 OMIM diseases with this phenotype, 10 are associated with *PIK3CA* and 9 are associated with *KRAS*. As a more specific example, the pair with the third highest score is between the gene *GLB1* and the HPO term *Decreased* β *-galactosidase activity*. This gene encodes the β -galactosidase gene, crucial for breaking down GM1 gangliosides. This gene is associated with four OMIM diseases [GM1-Gangliosidosis Type I (OMIM:230500), GM1-Gangliosidosis Type II (OMIM:230600), GM1-Gangliosidosis Type III (OMIM:230650), Mucopolisaccharidosis Type IVB (OMIM:253010)], all of which hold this phenotype. In fact, this phenotype is only displayed by these four diseases plus one other.

**TABLE 2:** Top phenotype-gene pairs according to the hypergeometric index, based on the OMIM dataset using the protein-target based methodology.

HPO Term ID	Term Name	Gene Entrez	Gene symbol	HyI
HP:0001428	Somatic Mutation	5290	*PIK3CA*	16.77
HP:0001428	Somatic Mutation	3845	*KRAS*	14.93
HP:0008166	Decreased β -galactosidase activity	2720	*GLB1*	13.00
HP:0004440	Coronal craniosynostosis	2263	*FGFR2*	12.66
HP:0003126	Low-molecular-weight proteinuria	1184	*CLCN5*	12.52
HP:0009737	Lisch nodules	4763	*NF1*	1,230
HP:0000590	Progressive external ophthalmoplegia	5428	*POLG*	11.80
HP:0000852	Pseudohypoparathyroidism	2778	*GNAS*	11.45
HP:0000114	Proximal tubulopathy	1184	*CLCN5*	11.18
HP:0003548	Subsarcolemmal accumulations of abnormally shaped mitochondria	5428	*POLG*	10.98
HP:0001839	Split foot	8626	*TP63*	10.68
HP:0001054	Numerous nevi	673	*BRAF*	10.61
HP:0007341	Diffuse swelling of cerebral white matter	220296	*HEPACAM*	10.53
HP:0000531	Corneal crystals	1497	*CTNS*	10.53
HP:0000166	Severe periodontitis	1075	*CTSC*	10.53
HP:0001284	Areflexia	4359	*MPZ*	10.50
HP:0008404	Nail dystrophy	1294	*COL7A1*	10.47
HP:0003689	Multiple mitochondrial DNA deletions	5428	*POLG*	10.42
HP:0008368	Tarsal synostosis	9241	*NOG*	10.00
HP: 0000926	Platyspondyly	1280	*COL2A1*	9.98

HPO, Human Phenotype Ontology; HyI, hypergeometric index.

The results for Orphanet are shown in Supplementary Table 1. The top association is between the HPO term *Milia* and the gene *COL7A1*. The gene is associated with 9 diseases, of which 8 hold this phenotype. The phenotype is found in a total of 21 Orphanet diseases. The results for Orphanet show repeated categories in the top 20, such as *Cyclopia*. This phenotype is associated with a range of important developmental genes. The prominence of high-score associations involving this phenotype is due to it being present in only 6 Orphanet diseases, but these 6 diseases are associated with many of the same genes.

For the domain-target workflow, the total numbers of phenotype-target pairs found are shown in Table 1 (Phenotype-FunFam Pairs). The top pairs in terms of score are shown in Table 3. The top associations are between the phenotypes related to sperm flagella abnormalities and the FunFam 3.40.50.300-ff-49, named *Dynein axonemal heavy chain 5*. Of the 19 genes associated with the phenotype *Absent sperm flagella* 5 encode proteins that contain this functional domain according to the CATH resource (Sillitoe et al., 2021).

**TABLE 3:** Top phenotype-FunFam pairs according to the hypergeometric index, based on the OMIM dataset using the domain-target based methodology.

HPO Term ID	Term Name	FunFam	HyI
HP:0032558	Absent sperm flagella	3.40.50.300-ff-49	12.96
HP:0032560	Coiled sperm flagella	3.40.50.300-ff-49	12.84
HP:0032559	Short sperm flagella	3.40.50.300-ff-49	12.84
HP:0032558	Absent sperm flagella	1.10.8.1220-ff-1	11.82
HP:0010817	Linear nevus sebaceous	3.40.50.300-ff-96	11.74
HP:0010815	Nevus Sebaceous	3.40.50.300-ff-96	11.74
HP:0001167	Abnormality of finger	3.40.50.300-ff-96	11.74
HP:0032560	Coiled sperm flagella	1.10.8.1220-ff-1	11.69
HP:0032559	Short sperm flagella	1.10.8.1220-ff-1	11.69
HP:0032558	Absent sperm flagella	3.40.50.300-ff-38	11.61
HP:0032560	Coiled sperm flagella	3.40.50.300-ff-38	11.48
HP:0032559	Short sperm flagella	3.40.50.300-ff-38	11.48
HP:0011073	Abnormality of dental color	3.40.50.300-ff-96	11.13
HP:0003795	Short middle phalanx of toe	3.30.200.20-ff-11	11.13
HP:0001780	Abnormality of toe	3.40.50.300-ff-96	11.13
HP:0000267	Cranial asymmetry	3.40.50.300-ff-96	11.13
HP:0002676	Cloverleaf skull	3.30.200.20-ff-11	10.74
HP:0003795	Short middle phalanx of toe	2.60.40.10-ff-20	10.53
HP:0003795	Short middle phalanx of toe	1.10.510.10-ff-7	10.53
HP:0006482	Abnormality of dental morphology	3.40.50.300-ff-96	10.43

HPO, Human Phenotype Ontology; FunFam, CATH functional family; HyI, hypergeometric index.

Multiple FunFams from the same superfamily were also associated with HPO term *Nevus sebaceous*, however this FunFam was named *KRAS proto-oncogene, GTPase*. *KRAS* mutations have been associated with nevus sebaceous in previous work (Groesser 2012). All three genes that are associated with this phenotype contain this FunFam according to CATH.

For the Orphanet analysis (Supplementary Table 2), the association with highest score was between 1.20.5.500-ff-1 and *Follicular hyperkeratosis*. This FunFam was in the CATH superfamily Single helix bin, and the FunFam is named *Keratin 23* – showing clear relevance for this phenotypes. This phenotype is associated with 13 genes, of which 7 encode proteins that contain this FunFam according to CATH (Sillitoe et al., 2021).

### 3.2 Drug-target pairs

For the protein-target based workflow, the drug-target pairs were obtained directly from ChEMBL as described in methods. This resulted in 264,690 drugs, which could be mapped to a total of 2,029 protein targets (367,934 drug-protein pairs). For the domain-target workflow, there was an extra step, whereby the drugs were mapped to FunFam domains, under the premise that a drug mapped to a number of proteins with the same domain is more likely to be interacting with that domain. This resulted in a total of 254,791 drugs/compounds that could be potentially associated with 3,420 domain targets (894,263 drug-domain pairs).

### 3.3 Drug-phenotype associations

Once we had obtained phenotype-gene pairs and the drug-target pairs we could combine them to associate drugs with phenotypes based on shared targets/genes. The total numbers of pairs, i.e., drugs linked to phenotypes via at least one shared target, and the numbers of pairs retained at different score thresholds are shown in Table 4 for the protein and domain-target workflows, respectively.

**TABLE 4:** Numbers of phenotype-drug pairs within the tripartite network and at different association thresholds for the protein-target workflow and for the domain-target workflow.

	Protein-target method					
	OMIM			Orphanet		
	Pairs	Phenotype	Drugs	Pairs	Phenotype	Drugs
Total	2,012,260	3,666	141,418	2,260,499	3,830	112,693
HyI ≥ 2	1,827,126	3,597	140,615	1,956,625	3,711	111,809
HyI ≥ 3	458,240	1,828	95,851	377,189	1,509	76,897
HyI ≥ 3.5	111,608	728	39,397	99,566	691	31,625

	Domain-Target Method					
	OMIM			Orphanet		
	Pairs	Phenotype	Drugs	Pairs	Phenotype	Drugs
Total	3,283,330	4,119	159,282	3,683,928	4,257	143,879
HyI ≥ 2	2,943,821	4,039	159,036	3,383,424	4,195	143,183
HyI ≥ 3	2,395,901	3,774	143,523	2,727,330	3,804	123,787
HyI ≥ 3.5	2,290,236	3,599	122,882	2,564,173	3,618	117,703

HyI, hypergeometric index.

In order to validate these results, we filtered them to only include drug-phenotype pairs for drugs and phenotypes that are annotated within the SIDER database. Only a small number of drugs from ChEMBL actually have any documented effects.

### 3.4 Overlap with known effects from the SIDER database

**FIGURE 2:**
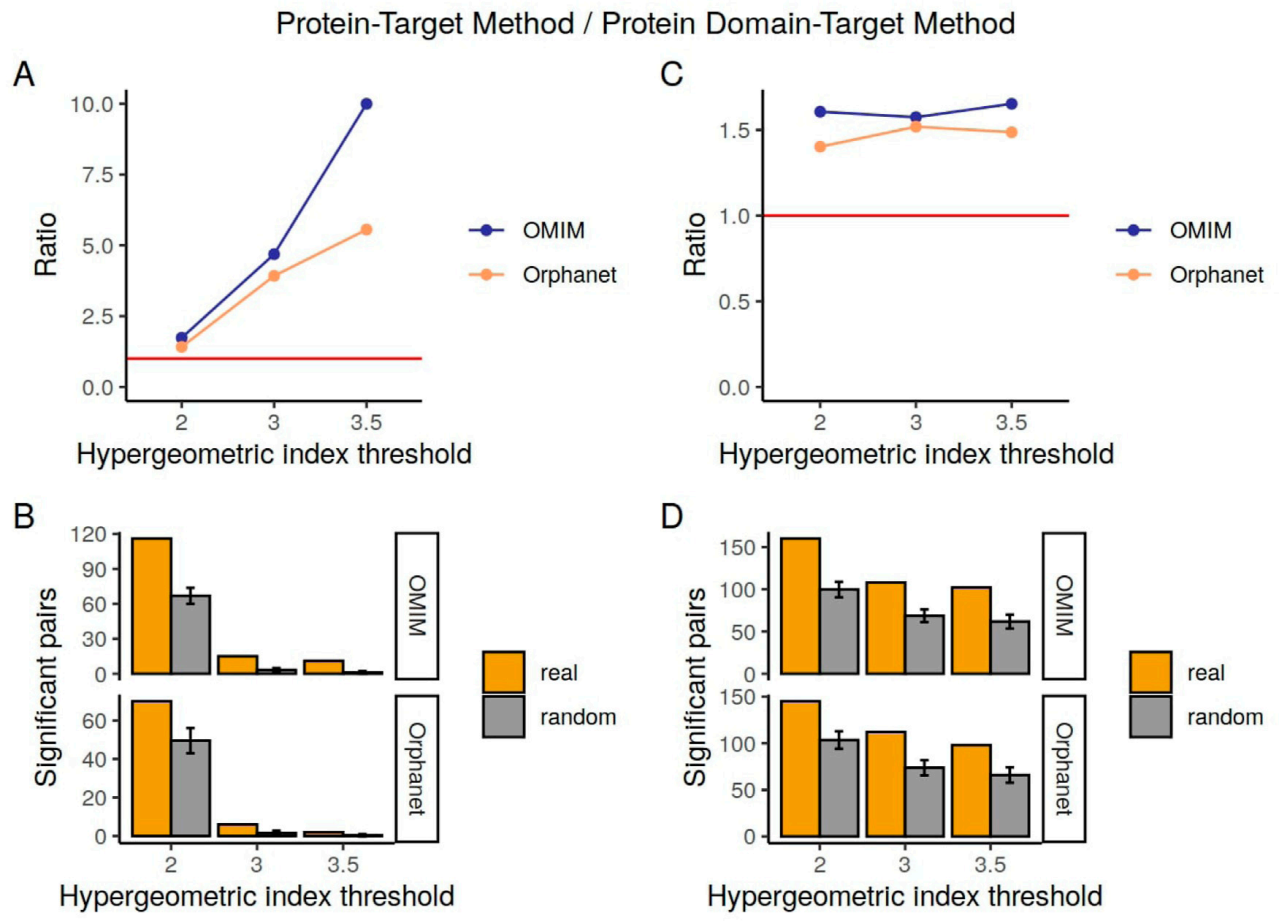
Numbers of pairs in the Confirmed Pairs lists from the SIDER database compared to the Random Overlapping lists. On the left are the pairs for the protein-target method and on the right the pairs for the domain-target method. **(A, C)** show the ratio changes in the different hypergeometric index thresholds. The red line corresponds to a ratio of 1. **(B, D)** represent the total number of pairs confirmed by SIDER per each hypergeometric index threshold.

Of the large number of phenotype-drug pairs obtained by our methods, the majority corresponded to drugs under early stages of development and as such there is unlikely to be any representation of the associated phenotype-drug pairs within SIDER due to 1) the lack of name for the drug (normally *NA* or potentially a compound) 2) because the drug has not passed through a sufficient number of stages of the development pipelines, its effects have not yet been detected or determined. Therefore, when we filtered the phenotype-drug pair results to only include those for which the drug and phenotype exist within SIDER, we retained comparatively small numbers of pairs. In Table 5, the numbers of pairs that overlap with known drug-effects from the SIDER database are shown in the column Confirmed Pairs, for different hypergeometric index thresholds. The column Random Overlapping shows the mean number of pairs found in the random pair lists, generated by shuffling connections between the phenotypes and drugs in the Confirmed Pairs list, that overlap with the known drug-effects. The ratio between the Confirmed Pairs and the Random Overlapping pairs is also shown. The numbers of pairs in the overlapping lists compared to the random lists are plotted in Figure 2 to make the trends clearer. For the protein-target results there is a clear increase in terms of performance, as measured by overlap with SIDER compared to random, with higher hypergeometric index thresholds. This trend appears more marked for OMIM than for Orphanet. For example, the number of predicted pairs confirmed by SIDER are between 1.54 and 10 times higher than what would have been obtained by chance for the protein-target approach with OMIM annotations. It is also clear that the total number of pairs obtained decreases with the increased threshold, again this is more marked for the protein-target method results.

**TABLE 5:** Overlap between the drug-phenotype pairs detected by the protein-target method, domain-target method and SIDER database.

	Protein-target method					
	OMIM			Orphanet		
	Confirmed pairs	Random overlapping	Ratio	Confirmed pairs	Random overlapping	Ratio
Total	280	181.39 ± 12.52	1.54	300	169.14 ± 11.16	1.77
HyI ≥ 2	116	66.84 ± 6.84	1.74	70	49.56 ± 6.54	1.41
HyI ≥ 3	15	3.20 ± 1.71	4.69	6	1.53 ± 1.23	3.92
HyI ≥ 3.5	11	1.10 ± 1.04	10	2	0.36 ± 0.61	5.56

	Domain-target method					
	OMIM			Orphanet		
	Confirmed pairs	Random overlapping	Ratio	Confirmed pairs	Random overlapping	Ratio
Total	255	152.98 ± 12.46	1.67	200	145.07 ± 11.11	1.38
HyI ≥ 2	160	99.61 ± 9.12	1.61	145	103.44 ± 9.37	1.40
HyI ≥ 3	108	68.60 ± 7.57	1.57	112	73.74 ± 8.15	1.52
HyI ≥ 3.5	102	61.72 ± 8.21	1.65	98	65.93 ± 8.21	1.49

HyI, hypergeometric index.

### 3.5 Overlap with drug-phenotype pairs obtained from the biomedical literature

**FIGURE 3:**
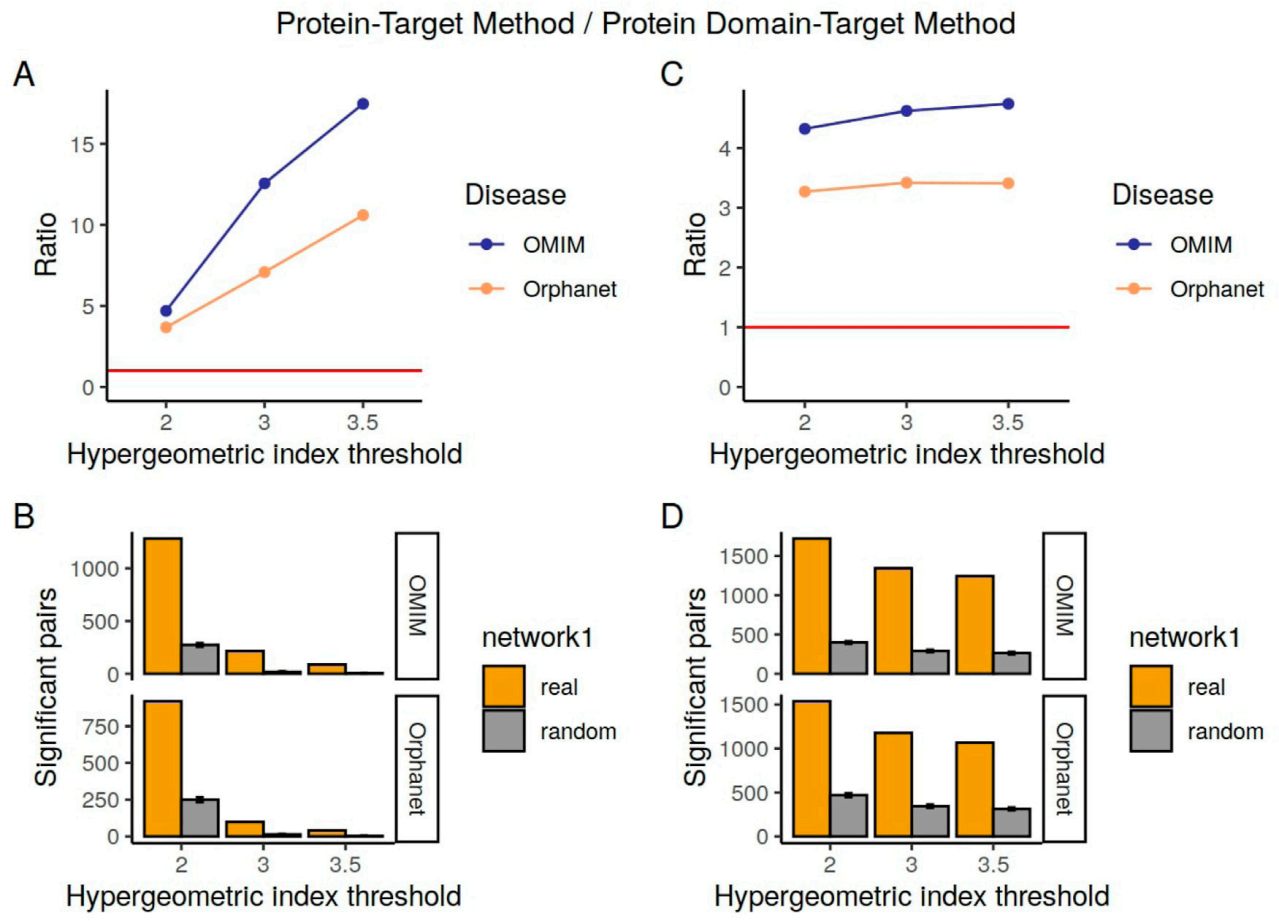
Numbers of pairs in the Confirmed Pairs lists from the literature derived co-occurrence data compared to the Random Overlapping lists. On the left are the pairs for the protein-target method and on the right the pairs for the domain-target method. **(A, C)** show the ratio changes in the different hypergeometric index thresholds. The red line corresponds to a ratio of 1. **(B, D)** represent the total number of pairs confirmed by SIDER per each hypergeometric index threshold.

As well as overlap with known drug-effects data from SIDER, we looked at the overlap between our phenotype-drug associations and the biomedical literature, in order to see whether our pairs are more likely to co-occur together in PubMed abstracts than they would if we were to randomize the connections. Results are shown in Table 6, for the protein-target and domain-target methods, and Figure 3.

**TABLE 6:** Overlap between the drug-phenotype pairs detected by the protein-target target method, domain-target method and the co-occurrence dataset.

	Protein-target method					
	OMIM			Orphanet		
	Confirmed pairs	Random overlapping	Ratio	Confirmed pairs	Random overlapping	Ratio
Total	2,111	570.52 ± 24.84	3.70	1,674	570.93 ± 20.08	2.93
HyI ≥ 2	1,283	273.48 ± 17.55	4.69	919	250.07 ± 16.81	3.67
HyI ≥ 3	215	17.12 ± 4.35	12.56	99	13.98 ± 3.84	7.08
HyI ≥ 3.5	87	4.98 ± 2.08	17.47	41	3.87 ± 1.86	10.59

	Domain-Target Method					
	OMIM			Orphanet		
	Confirmed pairs	Random overlapping	Ratio	Confirmed pairs	Random overlapping	Ratio
Total	2,127	532.94 ± 22.13	3.99	1,784	576.63 ± 19.16	3.09
HyI ≥ 2	1,721	398.24 ± 17.78	4.32	1,536	469.76 ± 22.57	3.27
HyI ≥ 3	1,342	290.34 ± 15.89	4.62	1,178	344.55 ± 18.39	3.42
HyI ≥ 3.5	1,243	262.20 ± 16.49	4.74	1,067	312.90 ± 16.31	3.41

HyI, hypergeometric index.

We see that our predicted associations are several times more likely to co-occur in abstracts than random, and that this tendency increases when a higher hypergeometric index threshold is used to determine the significantly associated drug-phenotype pairs. Although our methods find many more known drug-phenotype associations than random for both SIDER and the literature, both the total numbers of pairs found and the ratios compared to random are generally higher when benchmarked against the literature than for SIDER. This is likely to do with the limitation of SIDER in terms of only containing adverse effects for marketed drugs. In contrast, the co-occurrence method is able to find both the side effects and desired effects, and can potentially include drugs in different stages of development.

### 3.6 Drug-phenotype associations with the highest association values

The top 20 drug-phenotype associations are shown in Tables 7, 8 (OMIM results) and Supplementary Tables 3, 4 (Orphanet results). These binary associations are also presented as networks in Supplementary Figures 1, 2. For the protein-target based method (Table 7), applied to the OMIM database, the top result is for the drug nilotinib, associated with the phenotype *Chronic myelogenous leukemia*. Nilotinib is a medication used to treat chronic myelogenous leukemia. Clearly this is not an adverse effect, rather the pathology that the drug is intended to treat. The phenotype and drug are linked via the proteins encoded by genes *ABL1* and *BCR*. The next phenotype in the list is *Dry skin*, associated with the drug vemurafenib, used to treat melanoma. *Dry skin* is indeed listed as one of the side effects–interestingly, the association between this phenotype and the drug is mediated by the proteins coded for by genes *RAF1* and *BRAF*, both of which have been linked with atopic dermatitis in previous work (Raguz et al., 2016).

**TABLE 7:** Top drug-phenotype pairs according to the hypergeometric index, based on the OMIM dataset using the protein-target based methodology, only including ChEMBL drugs with drug names that can be found within SIDER (all drugs are in phase 4).

HPO Term ID	Term Name	Drug	Drug name	HyI	Evidence
HP:0005506	Chronic myelogenous leukemia	CHEMBL 255863	NILOTINIB	4.72	PMID: 30547682, PMID: 33414482
HP:0005506	Chronic myelogenous leukemia	CHEMBL 941	IMATINIB	4.65	PMID: 33414482, PMID: 24455116
HP:0000958	Dry skin	CHEMBL 1229517	VEMURAFENIB	4.56	PMID: 26328215, PMID: 27699043
HP:0005506	Chronic myelogenous leukemia	CHEMBL 288441	BOSUTINIB	4.24	PMID: 30446802, PMID: 39164407
HP:0005506	Chronic myelogenous leukemia	CHEMBL 1171837	PONATINIB	4.19	PMID: 38287132, PMID: 38804723
HP:0000746	Delusions	CHEMBL 589	ROPINIROLE	3.91	PMID: 21494343, PMID: 22953148
HP:0100723	Gastrointestinal stroma tumor	CHEMBL 941	IMATINIB	3.88	PMID: 38886160, PMID: 37254018
HP:0100753	Schizophrenia	CHEMBL 243712	AMISULPRIDE	3.84	PMID: 29406775, PMID: 12076408, PMID: 11803729
HP:0000746	Delusions	CHEMBL 243712	AMISULPRIDE	3.69	PMID: 12076408, PMID: 11803729
HP:0005506	Chronic myelogenous leukemia	CHEMBL 1421	DASATINIB	3.69	PMID: 19536317, PMID: 27784993
HP:0100723	Gastrointestinal stroma tumor	CHEMBL 477772	PAZOPANIB	3.51	PMID: 34271307
HP:0000958	Dry skin	CHEMBL 1946170	REGORAFENIB	3.41	PMID: 37666264
HP:0002019	Constipation	CHEMBL 669	CYCLOBENZ-APRINE	3.27	PMID: 20675978
HP:0000958	Dry skin	CHEMBL 2028663	DABRAFENIB	3.16	PMID: 37610803
HP:0011034	Amyloidosis	CHEMBL 2103837	TAFAMIDIS	3.15	PMID: 30145929 (Transthyretin Amyloid Cardiomyopathy)
HP:0000958	Dry skin	CHEMBL 477772	PAZOPANIB	2.97	PMID: 25031940, PMID: 25592338 (Skin reactions but not dry) skin
HP:0006721	Acute lymphoblastic leukemia	CHEMBL 941	IMATINIB	2.93	PMID: 38581291, PMID: 31944221, PMID: 21575924
HP:0100753	Schizophrenia	CHEMBL 71	CHLORPR-OMAZINE	2.89	PMID: 28407198
HP:0100753	Schizophrenia	CHEMBL 54	HALOPERIDOL	2.89	PMID: 31006114
HP:0000958	Dry skin	CHEMBL 1336	SORAFENIB	2.86	PMID: 22551785, PMID: 24698672

Drug, ChEMBL database ID; HyI, hypergeometric index.

**TABLE 8:** Top drug-phenotype pairs according to the hypergeometric index, based on the OMIM dataset using the domain-target based methodology, only including ChEMBL drugs with drug names that can be found within SIDER.

HPO Term ID	Term Name	Drug	Drug name	HyI	Evidence
HP:0005506	Chronic myelogenous leukemia	CHEMBL 255863	NILOTINIB	26.30	PMID: 30547682, PMID: 33414482
HP:0000622	Blurred vision	CHEMBL 698	TETRACAINE	26.22	PMID: 28521706, PMID: 33121832
HP:0005506	Chronic myelogenous leukemia	CHEMBL 941	IMATINIB	24.97	PMID: 33414482, PMID: 24455116
HP:0006721	Acute lymphoblastic leukemia	CHEMBL 941	IMATINIB	18.93	PMID: 38581291, PMID: 31944221, PMID: 21575924
HP:0006721	Acute lymphoblastic leukemia	CHEMBL 1171837	PONATINIB	17.54	PMID: 38828928, PMID: 39328803, PMID: 38972767
HP:0001269	Hemiparesis	CHEMBL 1908360	EVEROLIMUS	17.10	PMID: 28888335 (used in cancer treatment; immunosuppressive therapy, but also for seizures and epilepsy. mTOR inhibitor side effects can lead to hemiparesis in rare cases)
HP:0004936	Venous thrombosis	CHEMBL 1171837	PONATINIB	16.41	PMID: 32911643
HP:0100723	Gastrointestinal stroma tumor	CHEMBL 941	IMATINIB	15.24	PMID: 38886160, PMID: 37254018
HP:0100723	Gastrointestinal stroma tumor	CHEMBL 477772	PAZOPANIB	13.40	PMID: 34271307
HP:0002170	Intracranial hemorrhage	CHEMBL 231779	APIXABAN	12.71	PMID: 23220847, PMID: 27823792
HP:0000225	Gingival bleeding	CHEMBL 231779	APIXABAN	12.27	PMID: 26535102
HP:0000421	Epistaxis	CHEMBL 231779	APIXABAN	11.55	PMID: 23220847, PMID: 26535102
HP:0000132	Menorrhagia	CHEMBL 231779	APIXABAN	11.55	PMID: 23220847
HP:0005506	Chronic myelogenous leukemia	CHEMBL 288441	BOSUTINIB	11.27	PMID: 38278737
HP:0002019	Constipation	CHEMBL 669	CYCLOBENZ-APRINE	11.17	PMID: 20675978
HP:0001664	Torsade de pointes	CHEMBL 473	DOFETILIDE	10.80	PMID: 25634399, PMID: 39221117
HP:0001664	Torsade de pointes	CHEMBL 1108	DROPERIDOL	10.80	PMID: 19291568
HP:0001664	Torsade de pointes	CHEMBL 1008	BEPRIDIL	10.80	PMID: 33026317
HP:0002204	Pulmonary embolism	CHEMBL 48361	DABIGATRAN	10.68	PMID: 27411591
HP:0012531	Pain	CHEMBL 698	TETRACAINE	10.61	PMID: 31739347 (analgesic)

Drug, ChEMBL database ID; HyI, hypergeometric index.

The top results for Orphanet also show known effects, such as the association between the kinase inhibitor cabozantinib and nausea. The mechanism here is perhaps less clear, given that the connection is via the proteins coded by *KIT* and *RET*.

For the domain-target methodology applied to OMIM (Table 8), the top result is the same as for the protein-target method: *Chronic myelogenous leukemia* and nilotinib. For this methodology, the drug and phenotype are connected by multiple domains belonging to FunFams related to kinase activity or breakpoint cluster regions. The second pair, *blurred vision* associated with tetracaine, was not found in the top 20 using the protein-target method. These entities are linked via domains belonging to FunFams related to sodium channels. For Orphanet, the top result is for *thrombocytosis* associated with nilotinib, again via tyrosine kinase domains and breakpoint cluster region-related domain. Nilotinib has great affinity for the breakpoint cluster region Abelson murine leukemia (BCR-ABL) viral protooncogene (Cervantes and Mauro, 2011; DeRemer et al., 2008), which has an important role in the stimulation of growth and prevention of apoptosis in hematopoietic cells (Neshat et al., 2000), including platelets levels (Bennour et al., 2013), suggesting a potential mechanistic link. In fact, previous studies have looked at the relationship between nilotinib and platelet function (Alqasim et al., 2018).

The top 20 drug-phenotype associations for comention analysis are shown in Supplementary Tables 5, 6 for OMIM results and Supplementary Tables 7, 8 for Orphanet results.

## 4 Discussion

By combining drug-target and target-phenotype information from different sources, our approach generates an exhaustive list of putative drug effects. This allows us to identify known effects and to predict novel putative effects for drugs, including both unwanted adverse side effects and intended effects (i.e., the pathological phenotype the drug was designed to treat). In addition to these putative effects, our approach provides information on the involved genes and protein-domains, offering clues as to the molecular mechanisms underlying them.

These results reinforce the premise on which this study is based: the pathological phenotypes associated with variants in each protein can also occur when the protein is drugged. For loss-of-function mutations and drugs that inactivate or inhibit proteins, this tenet holds. However, in other cases, such as many cancers, the variants may lead to a gain of function. Moreover, whilst most drugs are deemed inhibitors, many are potentiators or activators (Wishart et al., 2018). As such, by combining drug-target and phenotype-target pairs, we also find links between drugs and phenotypes that are caused by the activation of the protein, rather than its inhibition. The clearest example of this is provided by the top results using both protein-target and domain-target methods, where we show a connection between *Chronic myelogenous leukemia* and nilotinib–these are actually linked by potential oncogenes (Sánchez-García and Grütz, 1995), which fits with the idea that it is the activation of the protein that leads to the phenotype, and therefore, their inhibition leads to the therapeutically intended effect against this type of cancer.

The methodology, by initially associating drugs with targets, and target with phenotypes, is intended to model how drugs lead to their effects. This has potential applications for excluding drugs with potential side-effects during the design process. For example, a drug that is known to target a protein or domain linked to a phenotype that would be particularly harmful in the intended patients could be considered for exclusion. Conversely, when this effect is the pathology that the drug was intended for, the methodology can provide the user with additional information about the mechanisms through which their drug works. Our results also have implications for drug repurposing, as some of the predicted drug effects could be of interest. There are many previous studies that attempt to link drugs with known diseases, often with the aim of repurposing existing drugs for different diseases (e.g., Sadegh et al., 2021; Lotfi Shahreza et al., 2020; Zhang et al., 2018; Lee and Yoon, 2018; Liu et al., 2016; Gottlieb et al., 2011). Whilst the aim of our method is quite different, the ability to obtain the intended effects of drugs based on the combination of drug-target and target-phenotype data means that it could potentially be used alongside these methods. However, it should be considered that the effects we find are not necessarily the desired ones. Further work could try to differentiate intended from adverse/side effects, for example, by stratifying drugs into different categories depending on their effects, and potentially by further refining the phenotype-gene data to consider mutation effects.

Although several studies look to detect drug-adverse/side effect interactions directly using the biomedical literature (Kropiwnicki et al., 2022; drissiya El-allaly et al., 2019; Song et al., 2019; Xu and Wang, 2015; Shang et al., 2014; Coulet et al., 2010), few seek to understand the interactions considering the protein drug targets and the phenotypes they may lead to based on genetic diseases network. Recent work (Nguyen et al., 2019) accumulated data to link 1,819 drugs with 1,046 targets to show how the genes encoding drug targets could be used to make predictions about clinical side effects, such as the organ system affected. This work has been fundamental in showing the validity of combining data from different sources. Our work differs from their approach in that we take all drug-protein information available in the ChEMBL database as starting material. It also differs in that it can consider protein domain families as well as genes when building the networks to link drugs, their targets, and potential side effects. As such it was able to model relationships for up to 159,282 different ChEMBL drugs with known protein targets. However, we must also point out that we were only able to perform validation analysis on a subset of drugs, as most of the ChEMBL drugs were at clinical trial phase 0 in the drug-development pipeline and have no official name other than their ChEMBL ID. As such, most of them have no documented effects according to the SIDER resource.

Although the protein-target and domain-target methods showed some similarity in terms of their results, the majority of the top twenty phenotype-drug associations were different. This is to be expected–one protein can be annotated with multiple domains, and a given domain might belong to multiple proteins. The domain-target method has the distinct advantage that it allows finding the putative domain involved in how drugs exert their phenotypic effects, providing information at a finer molecular detail than the protein-target approach. It also has the advantage of finding a larger number of significant associations at all hypergeometric index thresholds, for both OMIM and Orphanet diseases. Nevertheless, it should also be pointed out that the protein-target method tends to perform better than the domain-target method. This may be due to the sheer number of steps in the domain-target workflow, which contains extra steps in both modules A and B to map from proteins to domains, and we may be losing information here. By using FunFams we also potentially include noise by connecting drugs to multiple domain families, some of which might be connected to many different functions.

It is also clear that the OMIM results tend to show higher overlap with known associations than the Orphanet results. This is likely because OMIM contains Mendelian diseases, with each disease largely considered a separate entity. As such, the link between the variant, gene and phenotype is arguably more direct, whereas for Orphanet a given disease is more likely to be linked to multiple genes. In fact, the mean number of genes per disease in Orphanet is almost twice as high as for OMIM (2.19 vs. 1.12). Future work could explore the use of Orphanet data in a different way, combining the data from the different resources, or using resources such as MONDO/The Monarch initiative (Shefchek et al., 2020; Vasilevsky et al., 2022).

Whilst other metrics exist beside the hypergeometric index, we have shown in previous studies that it provides the best results when applied to similar data (Bueno et al., 2018; Rojano et al., 2017). Nevertheless, other studies have shown other metrics to perform well for different types of networks and this remains a potential avenue for future work. Another important line of research for the future is to differentiate drug-phenotype associations related to intended from those related to side/adverse effects, side-effects databases such as SIDER could potentially be exloited for this purpose. It would also be interesting to look at combining OMIM and Orphanet data, both at the level of network creation and in terms of overlapping results.

To conclude, we have developed two workflows to associate drugs with phenotypes based on combining drug-target and target-phenotype pairs, taken from disease and drug databases. We have shown that these phenotype-drug pairs show high overlap with drug-effects pairs taken from a database of known side effects and are frequently found together in the scientific literature. This adds weight to previous findings (Alqasim et al., 2018) involving the use of target data to understand drug effects. The results derived from this study could have a significant impact on drug development and repositioning. The tripartite network-based approach that links drugs, targets, and phenotypes, provides insights into the mechanisms that connect drugs to their potential effects, both intended and adverse side effects, as well as potential off-targets. The approach could help to identify potential outcomes at early stages of the development process, which can reduce failure rates, saving time and money. Moreover, the methodology may facilitate drug repurposing by identifying new therapeutic uses for existing drugs through shared targets and phenotypes.

## Data Availability

The original contributions presented in the study are included in the article/Supplementary Material, further inquiries can be directed to the corresponding author.

## Abbreviations

FunFam, Functional Family; HPO, Human Phenotype Ontology.

## References

[B1] Alqasim A. M. Z. Obaid G. M. Yaseen Y. G. Alwan A. F. (2018). Effects of nilotinib on platelet function in patients with chronic myeloid leukemia in chronic phase. Leukemia Res. Rep. 11, 46–50. 10.1016/J.LRR.2018.05.003 31293883 PMC6594045

[B2] Bass J. I. F. Diallo A. Nelson J. Soto J. M. Myers C. L. Walhout A. J. M. (2013). Using networks to measure similarity between genes: association index selection. Nat. Methods 10, 1169–1176. 10.1038/nmeth.2728 24296474 PMC3959882

[B3] Bennour A. Ouahchi I. Achour B. Zaier M. Youssef Y. B. Khelif A. (2013). Analysis of the clinico-hematological relevance of the breakpoint location within M-BCR in chronic myeloid leukemia. Med. Oncol. N. Lond. Engl. 30, 348. 10.1007/S12032-012-0348-Z 23269583

[B4] Bodenreider O. (2004). The unified Medical Language System (UMLS): integrating biomedical terminology. Nucleic Acids Res. 32, D267–D270. 10.1093/NAR/GKH061 14681409 PMC308795

[B5] Bremner J. D. (2021). Isotretinoin and neuropsychiatric side effects: continued vigilance is needed. J. Affect. Disord. Rep. 6, 100230. 10.1016/j.jadr.2021.100230 37168254 PMC10168661

[B6] Bueno A. Rodríguez-López R. Reyes-Palomares A. Rojano E. Corpas M. Nevado J. (2018). Phenotype-loci associations in networks of patients with rare disorders: application to assist in the diagnosis of novel clinical cases. Eur. J. Hum. Genet. 26, 1451–1461. 10.1038/s41431-018-0139-x 29946186 PMC6138686

[B7] Campillos M. Kuhn M. Gavin A. C. Jensen L. J. Bork P. (2008). Drug target identification using side-effect similarity. Sci. (New York, N.Y.) 321, 263–266. 10.1126/SCIENCE.1158140 18621671

[B8] Cervantes F. Mauro M. (2011). Practical management of patients with chronic myeloid leukemia. Cancer 117, 4343–4354. 10.1002/CNCR.26062 21413002

[B9] Chaudhari R. Fong L. W. Tan Z. Huang B. Zhang S. (2020). An up-to-date overview of computational polypharmacology in modern drug discovery. Expert Opin. drug Discov. 15, 1025–1044. 10.1080/17460441.2020.1767063 32452701 PMC7415563

[B10] Chaudhari R. Tan Z. Huang B. Zhang S. (2017). Computational polypharmacology: a new paradigm for drug discovery. Expert Opin. drug Discov. 12, 279–291. 10.1080/17460441.2017.1280024 28067061 PMC7241838

[B11] Coulet A. Shah N. H. Garten Y. Musen M. Altman R. B. (2010). Using text to build semantic networks for pharmacogenomics. J. Biomed. Inf. 43, 1009–1019. 10.1016/J.JBI.2010.08.005 PMC2991587 20723615

[B12] Czernichow S. Batty D. (2010). Withdrawal of sibutramine for weight loss: where does this leave clinicians? Obes. Facts 3, 155–156. 10.1159/000316508 20616603 PMC6452142

[B13] Das S. Scholes H. M. Sen N. Orengo C. (2021). CATH functional families predict functional sites in proteins. Bioinforma. Oxf. Engl. 37, 1099–1106. 10.1093/BIOINFORMATICS/BTAA937 PMC8150129 33135053

[B14] Davies M. Nowotka M. Papadatos G. Dedman N. Gaulton A. Atkinson F. (2015). ChEMBL web services: streamlining access to drug discovery data and utilities. Nucleic Acids Res. 43, W612–W620. 10.1093/NAR/GKV352 25883136 PMC4489243

[B15] Davis R. L. (2020). Mechanism of action and target identification: a matter of timing in drug discovery. iScience 23, 101487. 10.1016/j.isci.2020.101487 32891054 PMC7479624

[B16] Dawson N. Sillitoe I. Marsden R. L. Orengo C. A. (2017). The classification of protein domains. New York, NY: Springer, 137–164. 10.1007/978-1-4939-6622-6_7 27896721

[B17] DeRemer D. L. Ustun C. Natarajan K. (2008). Nilotinib: a second-generation tyrosine kinase inhibitor for the treatment of chronic myelogenous leukemia. Clin. Ther. 30, 1956–1975. 10.1016/J.CLINTHERA.2008.11.014 19108785

[B18] Díaz-Santiago E. Claros M. G. Yahyaoui R. de Diego-Otero Y. Calvo R. Hoenicka J. (2021). Decoding neuromuscular disorders using phenotypic clusters obtained from Co-occurrence networks. Front. Mol. Biosci. 8, 635074. 10.3389/fmolb.2021.635074 34046427 PMC8147726

[B19] Díaz-Santiago E. Jabato F. M. Rojano E. Seoane P. Pazos F. Perkins J. R. (2020). Phenotype-genotype comorbidity analysis of patients with rare disorders provides insight into their pathological and molecular bases. PLOS Genet. 16, e1009054. 10.1371/journal.pgen.1009054 33001999 PMC7553355

[B20] drissiya El-allaly E. Sarrouti M. En-Nahnahi N. Ouatik El Alaoui S. (2019). An adverse drug effect mentions extraction method based on weighted online recurrent extreme learning machine. Comput. methods programs Biomed. 176, 33–41. 10.1016/J.CMPB.2019.04.029 31200909

[B21] Estrada K. Froelich S. Wuster A. Bauer C. R. Sterling T. Clark W. T. (2021). Identifying therapeutic drug targets using bidirectional effect genes. Nat. Commun. 12, 2224. 10.1038/s41467-021-21843-8 33850126 PMC8044152

[B22] Furberg C. D. Pitt B. (2001). Withdrawal of cerivastatin from the world market. Curr. Control. Trials Cardiovasc. Med. 2, 205–207. 10.1186/CVM-2-5-205 11806796 PMC59524

[B23] Gottlieb A. Stein G. Y. Ruppin E. Sharan R. (2011). PREDICT: a method for inferring novel drug indications with application to personalized medicine. Mol. Syst. Biol. 7, 496. 10.1038/MSB.2011.26 21654673 PMC3159979

[B24] Hu G. Agarwal P. (2009). Human disease-drug network based on genomic expression profiles. PloS one 4, e6536. 10.1371/JOURNAL.PONE.0006536 19657382 PMC2715883

[B25] Huang H. Nguyen T. Ibrahim S. Shantharam S. Yue Z. Chen J. Y. (2015). DMAP: a connectivity map database to enable identification of novel drug repositioning candidates. BMC Bioinforma. 16 (Suppl. 1), S4. 10.1186/1471-2105-16-S13-S4 PMC4597058 26423722

[B26] Huang T. Lin K.-H. Machado-Vieira R. Soares J. C. Jiang X. Kim Y. (2023). Explainable drug side effect prediction via biologically informed graph neural network. medRxiv. 10.1101/2023.05.26.23290615 PMC13040068 41876492

[B27] Iwata H. Mizutani S. Tabei Y. Kotera M. Goto S. Yamanishi Y. (2013). Inferring protein domains associated with drug side effects based on drug-target interaction network. BMC Syst. Biol. 7 (Suppl. 6), S18. 10.1186/1752-0509-7-S6-S18 24565527 PMC4029543

[B28] Jarada T. N. Rokne J. G. Alhajj R. (2020). A review of computational drug repositioning: strategies, approaches, opportunities, challenges, and directions. J. cheminformatics 12, 46. 10.1186/S13321-020-00450-7 PMC7374666 33431024

[B29] Joshua Swamidass S. (2011). Mining small-molecule screens to repurpose drugs. Briefings Bioinforma. 12, 327–335. 10.1093/BIB/BBR028 21715466

[B30] Kabir A. Muth A. (2022). Polypharmacology: the science of multi-targeting molecules. Pharmacol. Res. 176, 106055. 10.1016/J.PHRS.2021.106055 34990865

[B31] Kinnings S. L. Liu N. Buchmeier N. Tonge P. J. Xie L. Bourne P. E. (2009). Drug discovery using chemical systems biology: repositioning the safe medicine Comtan to treat multi-drug and extensively drug resistant tuberculosis. PLoS Comput. Biol. 5, e1000423. 10.1371/JOURNAL.PCBI.1000423 19578428 PMC2699117

[B32] Köhler S. Gargano M. Matentzoglu N. Carmody L. C. Lewis-Smith D. Vasilevsky N. A. (2021). The human phenotype ontology in 2021. Nucleic acids Res. 49, D1207–D1217. 10.1093/NAR/GKAA1043 33264411 PMC7778952

[B33] Kropiwnicki E. Lachmann A. Clarke D. J. Xie Z. Jagodnik K. M. Ma’ayan A. (2022). DrugShot: querying biomedical search terms to retrieve prioritized lists of small molecules. BMC Bioinforma. 23, 76–16. 10.1186/s12859-022-04590-5 PMC8858480 35183110

[B34] Kruger F. A. Rostom R. Overington J. P. (2012). Mapping small molecule binding data to structural domains. BMC Bioinforma. 13 (Suppl. 1), S11. 10.1186/1471-2105-13-S17-S11 PMC3521243 23282026

[B35] Kuhn M. Letunic I. Jensen L. J. Bork P. (2016). The SIDER database of drugs and side effects. Nucleic Acids Res. 44, D1075–D1079. 10.1093/nar/gkv1075 26481350 PMC4702794

[B36] Lamb J. Crawford E. D. Peck D. Modell J. W. Blat I. C. Wrobel M. J. (2006). The Connectivity Map: using gene-expression signatures to connect small molecules, genes, and disease. Sci. (New York, N.Y.) 313, 1929–1935. 10.1126/SCIENCE.1132939 17008526

[B37] Lee S. Lee K. H. Song M. Lee D. (2011). Building the process-drug–side effect network to discover the relationship between biological Processes and side effects. BMC Bioinforma. 12 (2), S2–S12. 10.1186/1471-2105-12-S2-S2 PMC3073182 21489221

[B38] Lee T. Yoon Y. (2018). Drug repositioning using drug-disease vectors based on an integrated network. BMC Bioinforma. 19, 446. 10.1186/S12859-018-2490-X PMC6249928 30463505

[B39] Liu H. Song Y. Guan J. Luo L. Zhuang Z. (2016). Inferring new indications for approved drugs via random walk on drug-disease heterogenous networks. BMC Bioinforma. 17, 539. 10.1186/S12859-016-1336-7 PMC5259862 28155639

[B40] Li Wan Po A. Zhang W. Y. (1998). What lessons can be learnt from withdrawal of mibefradil from the market? Lancet 351, 1829–1830. 10.1016/S0140-6736(05)78800-0 9652662

[B41] Lotfi Shahreza M. Ghadiri N. Green J. R. (2020). A computational drug repositioning method applied to rare diseases: adrenocortical carcinoma. Sci. Rep. 2020 10 (1), 8846–8847. 10.1038/s41598-020-65658-x PMC7264316 32483162

[B42] Luo Q. Chen D. Fan X. Fu X. Ma T. Chen D. (2020). KRAS and PIK3CA bi-mutations predict a poor prognosis in colorectal cancer patients: a single-site report. Transl. Oncol. 13, 100874. 10.1016/J.TRANON.2020.100874 32947236 PMC7502368

[B43] Mendez D. Gaulton A. Bento A. P. Chambers J. De Veij M. Félix E. (2019). ChEMBL: towards direct deposition of bioassay data. Nucleic Acids Res. 47, D930-D940–D940. 10.1093/NAR/GKY1075 30398643 PMC6323927

[B44] Moya-García A. Adeyelu T. Kruger F. A. Dawson N. L. Lees J. G. Overington J. P. (2017). Structural and functional view of polypharmacology. Sci. Rep. 7, 10102–10114. 10.1038/s41598-017-10012-x 28860623 PMC5579063

[B45] Moya-García A. A. Ranea J. A. G. (2013). Insights into polypharmacology from drug-domain associations. Bioinformatics 29, 1934–1937. 10.1093/bioinformatics/btt321 23740740

[B46] Neshat M. S. Raitano A. B. Wang H.-G. Reed J. C. Sawyers C. L. (2000). The survival function of the bcr-abl oncogene is mediated by bad-dependent and -independent pathways: roles for phosphatidylinositol 3-kinase and raf. Mol. Cell. Biol. 20, 1179–1186. 10.1128/MCB.20.4.1179-1186.2000 10648603 PMC85238

[B47] Nguyen P. A. Born D. A. Deaton A. M. Nioi P. Ward L. D. (2019). Phenotypes associated with genes encoding drug targets are predictive of clinical trial side effects. Nat. Commun. 10, 1579. 10.1038/S41467-019-09407-3 30952858 PMC6450952

[B48] Onakpoya I. J. Heneghan C. J. Aronson J. K. (2016). Post-marketing withdrawal of 462 medicinal products because of adverse drug reactions: a systematic review of the world literature. BMC Med. 14, 10–11. 10.1186/s12916-016-0553-2 26843061 PMC4740994

[B49] Palomba G. Colombino M. Contu A. Massidda B. Baldino G. Pazzola A. (2012). Prevalence of KRAS, BRAF, and PIK3CA somatic mutations in patients with colorectal carcinoma may vary in the same population: clues from Sardinia. J. Transl. Med. 10, 178–179. 10.1186/1479-5876-10-178 22931052 PMC3480926

[B50] Pazos F. Chagoyen M. Seoane P. Ranea J. A. (2022). CoMent: relationships between biomedical concepts inferred from the scientific literature. J. Mol. Biol. 167568doi, 167568. 10.1016/j.jmb.2022.167568 35662459

[B51] Plenge R. M. (2016). Disciplined approach to drug discovery and early development. Sci. Transl. Med. 8, 349ps15. 10.1126/SCITRANSLMED.AAF2608 27464747

[B52] Raguz J. Jeric I. Niault T. Nowacka J. D. Kuzet S. E. Rupp C. (2016). Epidermal RAF prevents allergic skin disease. eLife 5, e14012. 10.7554/ELIFE.14012 27431613 PMC4951198

[B53] Rojano E. Seoane P. Bueno-Amoros A. Perkins J. R. Garcia-Ranea J. A. (2017). “Revealing the relationship between human genome regions and pathological phenotypes through network analysis,” in Lecture notes in computer science (including subseries lecture notes in artificial intelligence and lecture notes in bioinformatics), 197–207.

[B54] Rukov J. L. Wilentzik R. Jaffe I. Vinther J. Shomron N. (2014). Pharmaco-miR: linking microRNAs and drug effects. Briefings Bioinforma. 15, 648–659. 10.1093/BIB/BBS082 PMC4103536 23376192

[B55] Sadegh S. Skelton J. Anastasi E. Bernett J. Blumenthal D. B. Galindez G. (2021). Network medicine for disease module identification and drug repurposing with the NeDRex platform. Nat. Commun. 12 (1), 6848–6912. 10.1038/s41467-021-27138-2 34824199 PMC8617287

[B56] Sánchez-García I. Grütz G. (1995). Tumorigenic activity of the BCR-ABL oncogenes is mediated by BCL2. Proc. Natl. Acad. Sci. U. S. A. 92, 5287–5291. 10.1073/PNAS.92.12.5287 7777499 PMC41679

[B57] Seoane P. Ocaña S. Carmona R. Bautista R. Madrid E. M. Torres A. (2016). AutoFlow, a versatile workflow engine illustrated by assembling an optimised *de novo* transcriptome for a non-model species, such as faba bean (Vicia faba). Curr. Bioinforma. 11, 440–450. 10.2174/1574893611666160212235117

[B58] Shang N. Xu H. Rindflesch T. C. Cohen T. (2014). Identifying plausible adverse drug reactions using knowledge extracted from the literature. J. Biomed. Inf. 52, 293–310. 10.1016/J.JBI.2014.07.011 PMC4261011 25046831

[B59] Sharav Y. Benoliel R. (2008). Pharmacotherapy of acute orofacial pain. Orofac. Pain Headache, 349–376doi. 10.1016/B978-0-7234-3412-2.10015-X

[B60] Shefchek K. A. Harris N. L. Gargano M. Matentzoglu N. Unni D. Brush M. (2020). The Monarch Initiative in 2019: an integrative data and analytic platform connecting phenotypes to genotypes across species. Nucleic Acids Res. 48, D704-D715–D715. 10.1093/nar/gkz997 31701156 PMC7056945

[B61] Sillitoe I. Bordin N. Dawson N. Waman V. P. Ashford P. Scholes H. M. (2020). Cath: increased structural coverage of functional space. Nucleic Acids Res. 49, D266–D273. 10.1093/nar/gkaa1079 PMC7778904 33237325

[B62] Sillitoe I. Bordin N. Dawson N. Waman V. P. Ashford P. Scholes H. M. (2021). CATH: increased structural coverage of functional space. Nucleic Acids Res. 49, D266–D273. 10.1093/nar/gkaa1079 33237325 PMC7778904

[B63] Sirota M. Dudley J. T. Kim J. Chiang A. P. Morgan A. A. Sweet-Cordero A. (2011). Discovery and preclinical validation of drug indications using compendia of public gene expression data. Sci. Transl. Med. 3, 96ra77. 10.1126/scitranslmed.3001318 PMC3502016 21849665

[B64] Song M. Baek S. H. Heo G. E. Lee J. H. (2019). Inferring drug-protein–side effect relationships from biomedical text. Genes 10, 159. 10.3390/GENES10020159 30791472 PMC6409686

[B65] Sultana J. Cutroneo P. Trifirò G. (2013). Clinical and economic burden of adverse drug reactions. J. Pharmacol. Pharmacother. 4, S73–S77. 10.4103/0976-500X.120957 24347988 PMC3853675

[B66] Vasilevsky N. A. Matentzoglu N. A. Toro S. Flack J. E. Hegde H. Unni D. R. (2022). Mondo: unifying diseases for the world, by the world. medRxiv. 10.1101/2022.04.13.22273750

[B67] Wang Y. Chen S. Deng N. Wang Y. (2013). Drug repositioning by kernel-based integration of molecular structure, molecular activity, and phenotype data. PloS one 8, e78518. 10.1371/JOURNAL.PONE.0078518 24244318 PMC3823875

[B68] Wang Y. Y. Nacher J. C. Zhao X. M. (2012). Predicting drug targets based on protein domains. Mol. Biosyst. 8, 1528–1534. 10.1039/C2MB05450G 22402667

[B69] Wishart D. S. Feunang Y. D. Guo A. C. Lo E. J. Marcu A. Grant J. R. (2018). DrugBank 5.0: a major update to the DrugBank database for 2018. Nucleic acids Res. 46, D1074-D1082–D1082. 10.1093/NAR/GKX1037 29126136 PMC5753335

[B70] Wouters O. J. McKee M. Luyten J. (2020). Estimated research and development investment needed to bring a new medicine to market, 2009-2018. JAMA 323, 844–853. 10.1001/JAMA.2020.1166 32125404 PMC7054832

[B71] Xu R. Wang Q. Q. (2015). Large-scale automatic extraction of side effects associated with targeted anticancer drugs from full-text oncological articles. J. Biomed. Inf. 55, 64–72. 10.1016/J.JBI.2015.03.009 PMC4582661 25817969

[B72] Yang J. Li Z. Fan X. Cheng Y. (2014). Drug-disease association and drug-repositioning predictions in complex diseases using causal inference-probabilistic matrix factorization. J. Chem. Inf. Model. 54, 2562–2569. 10.1021/CI500340N 25116798

[B73] Yang L. Agarwal P. (2011). Systematic drug repositioning based on clinical side-effects. PloS one 6, e28025. 10.1371/JOURNAL.PONE.0028025 22205936 PMC3244383

[B74] Ye H. Liu Q. Wei J. (2014). Construction of drug network based on side effects and its application for drug repositioning. PloS one 9, e87864. 10.1371/JOURNAL.PONE.0087864 24505324 PMC3913703

[B75] Zeng X. Zhu S. Liu X. Zhou Y. Nussinov R. Cheng F. (2019). deepDR: a network-based deep learning approach to *in silico* drug repositioning. Bioinforma. Oxf. Engl. 35, 5191–5198. 10.1093/BIOINFORMATICS/BTZ418 PMC6954645 31116390

[B76] Zhang W. Yue X. Huang F. Liu R. Chen Y. Ruan C. (2018). Predicting drug-disease associations and their therapeutic function based on the drug-disease association bipartite network. Methods (San Diego, Calif.) 145, 51–59. 10.1016/J.YMETH.2018.06.001 29879508

[B77] Zhou W. Wang Y. Lu A. Zhang G. (2016). Systems pharmacology in small molecular drug discovery. Int. J. Mol. Sci. 17, 246. 10.3390/ijms17020246 26901192 PMC4783977

