# Supplementary material for: Better understanding the phenotypic effects of drugs through shared targets in genetic disease networks
Source: Front Pharmacol. 2025 Jan 22;15:1470931. doi: 10.3389/fphar.2024.1470931 (PMC11794328; doi:10.3389/fphar.2024.1470931)
Supplement: Supplementary file 1 [file DataSheet7.pdf]

Supp Table 7 Top drug-phenotype pairs according to the hypergeometric index, based on the Orphanet dataset using the protein-target based methodology, only including ChEMBL drugs with drug names found by literature comention Drug: ChEMBL database ID, HyI: hypergeometric index.

HPO	HPO name	Drug	Drug Name	Phase	HyI
HP:0011739	Dexamethasone-suppressible primary hyperaldosteronism	CHEMBL934	METYRAPONE	4	7.03
HP:0031475	Status epilepticus without prominent motor symptoms	CHEMBL507974	TETRODOTOXIN	3	6.72
HP:0011746	Secretory adrenocortical adenoma	CHEMBL934	METYRAPONE	4	6.55
HP:0025101	Dysgenesis of the hippocampus	CHEMBL507974	TETRODOTOXIN	3	6.13
HP:0000061	Ambiguous genitalia, female	CHEMBL9298	FADROZOLE	0	5.77
HP:0001688	Sinus bradycardia	CHEMBL45816	MIBEFRADIL	4	5.69
HP:0001664	Torsade de pointes	CHEMBL45816	MIBEFRADIL	4	5.62
HP:0008221	Adrenal hyperplasia	CHEMBL934	METYRAPONE	4	5.29
HP:0011746	Secretory adrenocortical adenoma	CHEMBL157101	KETOCONAZOLE	4	5.11
HP:0002121	Generalized non-motor (absence) seizure	CHEMBL3809595	NA	0	4.90
HP:0012125	Prostate cancer	CHEMBL191334	NUTLIN-3	0	4.80
HP:0008151	Prolonged prothrombin time	CHEMBL512351	BETRIXABAN	4	4.75
HP:0008151	Prolonged prothrombin time	CHEMBL206335	RAZAXABAN	0	4.75
HP:0002018	Nausea	CHEMBL276711	SEMAXANIB	3	4.71
HP:0002069	Bilateral tonic-clonic seizure	CHEMBL3809595	NA	0	4.59
HP:0002018	Nausea	CHEMBL1289926	AXITINIB	4	4.14
HP:0001912	Abnormal basophil morphology	CHEMBL941	IMATINIB	4	4.01
HP:0002121	Generalized non-motor (absence) seizure	CHEMBL507974	TETRODOTOXIN	3	3.93
HP:0008221	Adrenal hyperplasia	CHEMBL157101	KETOCONAZOLE	4	3.84
HP:0004319	Decreased circulating aldosterone level	CHEMBL157101	KETOCONAZOLE	4	3.77
