# Supplementary material for: Better understanding the phenotypic effects of drugs through shared targets in genetic disease networks
Source: Front Pharmacol. 2025 Jan 22;15:1470931. doi: 10.3389/fphar.2024.1470931 (PMC11794328; doi:10.3389/fphar.2024.1470931)
Supplement: Supplementary file 2 [file DataSheet2.pdf]

Supp Table 2 Top phenotype-FunFam pairs according to the hypergeometric index, based on the Orphanet dataset using the domain-target based methodology. HPO: Human Phenotype Ontology, FunFam: CATH functional family, HyI: hypergeometric index.

HPO	HPO name	FunFam	HyI
HP:0007502	Follicular hyperkeratosis	1.20.5.500-ff-1	13.53
HP:0002384	Focal impaired awareness seizure	1.20.120.350-ff-5	11.89
HP:0002384	Focal impaired awareness seizure	1.10.287.70-ff-6	11.89
HP:0002384	Focal impaired awareness seizure	1.20.120.350-ff-4	11.59
HP:0002384	Focal impaired awareness seizure	1.20.120.350-ff-3	11.59
HP:0002217	Slow-growing hair	1.20.5.500-ff-1	11.38
HP:0002384	Focal impaired awareness seizure	1.10.287.70-ff-4	11.33
HP:0002384	Focal impaired awareness seizure	1.10.287.70-ff-3	11.33
HP:0002384	Focal impaired awareness seizure	1.10.287.70-ff-1	11.33
HP:0002384	Focal impaired awareness seizure	1.10.238.10-ff-2	11.33
HP:0002217	Slow-growing hair	1.20.5.170-ff-4	11.22
HP:0007446	Palmoplantar blistering	1.20.5.500-ff-1	11.15
HP:0002384	Focal impaired awareness seizure	1.20.120.350-ff-2	11.11
HP:0000798	Oligozoospermia	3.30.70.330-ff-180	11.09
HP:0007502	Follicular hyperkeratosis	1.20.5.170-ff-4	11.01
HP:0002217	Slow-growing hair	1.20.5.1160-ff-1	10.47
HP:0007502	Follicular hyperkeratosis	1.20.5.1160-ff-1	10.26
HP:0000139	Uterine prolapse	2.60.120.1000-ff-1	9.71
HP:0011961	Non-obstructive azoospermia	3.30.70.330-ff-180	9.66
HP:0030268	Hyperplastic callus formation	1.20.5.500-ff-1	9.53
