# Supplementary material for: Better understanding the phenotypic effects of drugs through shared targets in genetic disease networks
Source: Front Pharmacol. 2025 Jan 22;15:1470931. doi: 10.3389/fphar.2024.1470931 (PMC11794328; doi:10.3389/fphar.2024.1470931)
Supplement: Supplementary file 3 [file DataSheet4.pdf]

*Supp Table 4 Top drug-phenotype pairs according to the hypergeometric index, based on the Orphanet dataset using the domain-target based methodology, only including ChEMBL drugs with drug names that can be found within SIDER. Drug: ChEMBL database ID, HyI: hypergeometric index*

| HPO | HPO name | Drug | Drug Name | HyI | Evidence |
|---|---|---|---|---|---|
| HP:0001894 | Thrombocytosis | CHEMBL255863 | NILOTINIB | 13.79 | PMID: 28761360, PMID: 37686630 |
| HP:0003401 | Paresthesia | CHEMBL1825138 | NA | 12.82 | |
| HP:0001894 | Thrombocytosis | CHEMBL941 | IMATINIB | 12.49 | PMID: 28761360, PMID: 37686630 |
| HP:0000132 | Menorrhagia | CHEMBL231779 | APIXABAN | 11.17 | PMID: 23220847 |
| HP:0002170 | Intracranial hemorrhage | CHEMBL48361 | DABIGATRAN | 10.98 | |
| HP:0000225 | Gingival bleeding | CHEMBL231779 | APIXABAN | 10.97 | PMID: 26535102 |
| HP:0100242 | Sarcoma | CHEMBL477772 | PAZOPANIB | 10.89 | PMID: 31123606 |
| HP:0002018 | Nausea | CHEMBL669 | CYCLOBENZAPRINE | 10.83 | PMID: 26425251 |
| HP:0001962 | Palpitations | CHEMBL669 | CYCLOBENZAPRINE | 10.56 | PMID: 739852 |
| HP:0012086 | Abnormal urinary color | CHEMBL58 | MITOXANTRONE | 9.35 | PMID: 15623667 |
| HP:0100749 | Chest pain | CHEMBL669 | CYCLOBENZAPRINE | 9.02 | |
| HP:0005214 | Intestinal obstruction | CHEMBL941 | IMATINIB | 9.02 | PMID: 12600228 |
| HP:0100723 | Gastrointestinal stroma tumor | CHEMBL941 | IMATINIB | 8.95 | PMID: 38886160, PMID: 37254018 |
| HP:0001688 | Sinus bradycardia | CHEMBL1008 | BEPRIDIL | 8.89 | PMID: 19359813 |
| HP:0001664 | Torsade de pointes | CHEMBL473 | DOFETILIDE | 8.83 | PMID: 25634399, PMID: 39221117 |
| HP:0001664 | Torsade de pointes | CHEMBL1108 | DROPERIDOL | 8.83 | PMID: 19291568 |
| HP:0001664 | Torsade de pointes | CHEMBL1008 | BEPRIDIL | 8.83 | PMID: 33026317 |
| HP:0005110 | Atrial fibrillation | CHEMBL549 | CITALOPRAM | 8.65 | PMID: 11793617 Induce bradycardia |
| HP:0005110 | Atrial fibrillation | CHEMBL473 | DOFETILIDE | 8.65 | PMID: 11568824 |
| HP:0005110 | Atrial fibrillation | CHEMBL43 | AMSACRINE | 8.65 | PMID: 38244650 |
