# Supplementary material for: Better understanding the phenotypic effects of drugs through shared targets in genetic disease networks
Source: Front Pharmacol. 2025 Jan 22;15:1470931. doi: 10.3389/fphar.2024.1470931 (PMC11794328; doi:10.3389/fphar.2024.1470931)
Supplement: Supplementary file 4 [file DataSheet6.pdf]

Supp Table 6 Top drug-phenotype pairs according to the hypergeometric index, based on the OMIM dataset using the domain-target based methodology, only including ChEMBL drugs with drug names found by literature comention Drug: ChEMBL database ID, HyI: hypergeometric index.

HPO	HPO name	Drug	Drug Name	Phase	HyI
HP:0030078	Lung adenocarcinoma	CHEMBL2347958	NA	0	37.89
HP:0033124	Increased serum sorbitol concentration	CHEMBL1467	ALLOPURINOL	4	34.87
HP:0100723	Gastrointestinal stroma tumor	CHEMBL1642	IMATINIB MESYLATE	4	34.77
HP:0004758	Effort-induced polymorphic ventricular tachycardia	CHEMBL2440854	NA	0	34.21
HP:0032147	Erythromelalgia	CHEMBL4208190	NA	0	33.77
HP:0007215	Periodic hyperkalemic paralysis	CHEMBL4208190	NA	0	33.77
HP:0033122	Absent P wave	CHEMBL3707392	ELECLAZINE	3	32.73
HP:0200026	Ocular pain	CHEMBL698	TETRACAINE	4	32.73
HP:0030078	Lung adenocarcinoma	CHEMBL2110732	DACOMITINIB	4	31.18
HP:0002069	Bilateral tonic-clonic seizure	CHEMBL4208190	NA	0	30.77
HP:0002069	Bilateral tonic-clonic seizure	CHEMBL3809595	NA	0	30.77
HP:0030078	Lung adenocarcinoma	CHEMBL1173655	AFATINIB	4	30.08
HP:0033258	Sudden unexpected death in epilepsy	CHEMBL4208190	NA	0	29.87
HP:0031475	Status epilepticus without prominent motor symptoms	CHEMBL4208190	NA	0	28.81
HP:0002133	Status epilepticus	CHEMBL4208190	NA	0	28.81
HP:0032147	Erythromelalgia	CHEMBL507974	TETRODOTOXIN	3	28.51
HP:0007215	Periodic hyperkalemic paralysis	CHEMBL507974	TETRODOTOXIN	3	28.51
HP:0100723	Gastrointestinal stroma tumor	CHEMBL276711	SEMAXANIB	3	28.47
HP:0003768	Periodic paralysis	CHEMBL4208190	NA	0	27.96
HP:0100723	Gastrointestinal stroma tumor	CHEMBL101253	VATALANIB	3	27.71
