# Supplementary material for: Better understanding the phenotypic effects of drugs through shared targets in genetic disease networks
Source: Front Pharmacol. 2025 Jan 22;15:1470931. doi: 10.3389/fphar.2024.1470931 (PMC11794328; doi:10.3389/fphar.2024.1470931)
Supplement: Supplementary file 5 [file DataSheet3.pdf]

Supp Table 3 Top drug-phenotype pairs according to the hypergeometric index, based on the Orphanet dataset using the protein-target based methodology, only including ChEMBL drugs with drug names that can be found within SIDER (all drugs are in phase 4). Drug: ChEMBL database ID, HyI: hypergeometric index

HPO	HPO name	Drug	Drug Name	HyI	Evidence
HP:0002018	Nausea	CHEMBL2105717	CABOZANTINIB	3.75	PMID: 38828446
HP:0002018	Nausea	CHEMBL1946170	REGORAFENIB	3.56	PMID: 36798653
HP:0002018	Nausea	CHEMBL477772	PAZOPANIB	3.12	PMID: 31630333, PMID: 28506529
HP:0000029	Testicular atrophy	CHEMBL1274	NILUTAMIDE	3.06	PMID: 8997470 It isn't reported as an adverse effect. The drug is used for prostate cancer because it that block the effects of androgens at their receptor sites. The lack of testosterone binding to their receptors can cause testicular atrophy
HP:0002018	Nausea	CHEMBL1171837	PONATINIB	3.02	Cancer treatment usually cause nausea or vomit
HP:0002018	Nausea	CHEMBL1336	SORAFENIB	3.00	PMID: 38828446
HP:0002018	Nausea	CHEMBL24828	VANDETANIB	2.97	PMID: 27711083, PMID: 27843809
HP:0002018	Nausea	CHEMBL669	CYCLOBENZAPRINE	2.97	PMID: 16197668
HP:0001962	Palpitations	CHEMBL669	CYCLOBENZAPRINE	2.82	PMID: 739852
HP:0001271	Polyneuropathy	CHEMBL2103837	TAFAMIDIS	2.76	PMID: 27878441
HP:0002310	Orofacial dyskinesia	CHEMBL1373	MODAFINIL	2.71	PMID: 21037146
HP:0100749	Chest pain	CHEMBL669	CYCLOBENZAPRINE	2.67	
HP:0002354	Memory impairment	CHEMBL135400	ZOPICLONE	2.62	PMID: 11062863, PMID: 38149178
HP:0002018	Nausea	CHEMBL1421	DASATINIB	2.53	PMID: 28566209, PMID: 27267844
HP:0007185	Loss of consciousness	CHEMBL317052	REGADENOSON	2.52	PMID: 37337782
HP:0000132	Menorrhagia	CHEMBL231779	APIXABAN	2.52	PMID: 23220847
HP:0001337	Tremor	CHEMBL135400	ZOPICLONE	2.49	PMID: 18077750
HP:0000771	Gynecomastia	CHEMBL806	FLUTAMIDE	2.49	PMID: 25270521, PMID: 16845534
HP:0000771	Gynecomastia	CHEMBL409	BICALUTAMIDE	2.49	PMID: 25270521 ,PMID: 16845534
HP:0000771	Gynecomastia	CHEMBL1274	NILUTAMIDE	2.49	PMID: 25270521, PMID: 16845534
