# Supplementary material for: Better understanding the phenotypic effects of drugs through shared targets in genetic disease networks
Source: Front Pharmacol. 2025 Jan 22;15:1470931. doi: 10.3389/fphar.2024.1470931 (PMC11794328; doi:10.3389/fphar.2024.1470931)
Supplement: Supplementary file 6 [file DataSheet1.pdf]

Supp Table 1 Top phenotype-gene pairs according to the hypergeometric index, based on the Orphanet dataset using the protein-target based methodology. HPO: Human Phenotype Ontology, HyI: hypergeometric index.

HPO	HPO name	Gene Entrez	Gene Symbol	HyI
**HP:0001056**	Milia	1294	*COL7A1*	16.10
**HP:0001030**	Fragile skin	1294	*COL7A1*	13.64
**HP:0009914**	Cyclopia	8928	*FOXH1*	13.22
**HP:0009914**	Cyclopia	84976	*DISP1*	13.22
**HP:0009914**	Cyclopia	7546	*ZIC2*	13.22
**HP:0009914**	Cyclopia	7050	*TGIF1*	13.22
**HP:0009914**	Cyclopia	6997	*CRIPTO*	13.22
**HP:0009914**	Cyclopia	6496	*SIX3*	13.22
**HP:0009914**	Cyclopia	4838	*NODAL*	13.22
**HP:0009914**	Cyclopia	2619	*GAS1*	13.22
**HP:0003798**	Nemaline bodies	4703	*NEB*	13.03
**HP:0100245**	Desmoid tumors	324	*APC*	12.68
**HP:0000871**	Panhypopituitarism	8928	*FOXH1*	12.68
**HP:0000871**	Panhypopituitarism	84976	*DISP1*	12.68
**HP:0000871**	Panhypopituitarism	7546	*ZIC2*	12.68
**HP:0000871**	Panhypopituitarism	7050	*TGIF1*	12.68
**HP:0000871**	Panhypopituitarism	6997	*CRIPTO*	12.68
**HP:0000871**	Panhypopituitarism	6496	*SIX3*	12.68
**HP:0000871**	Panhypopituitarism	4838	*NODAL*	12.68
**HP:0000871**	Panhypopituitarism	2619	*GAS1*	12.68
