# Supplementary material for: Better understanding the phenotypic effects of drugs through shared targets in genetic disease networks
Source: Front Pharmacol. 2025 Jan 22;15:1470931. doi: 10.3389/fphar.2024.1470931 (PMC11794328; doi:10.3389/fphar.2024.1470931)
Supplement: Supplementary file 7 [file DataSheet5.pdf]

*Supp Table 5 Top drug-phenotype pairs according to the hypergeometric index, based on the OMIM dataset using the protein-target based methodology, only including ChEMBL drugs with drug names found by literature comention Drug: ChEMBL database ID, HyI: hypergeometric index.*

| HPO | HPO name | Drug | Drug Name | Phase | HyI |
|---|---|---|---|---|---|
| HP:0002930 | Impaired sensitivity to thyroid hormone | CHEMBL557 | DETROTHYRONINE | 0 | 7.20 |
| HP:0002930 | Impaired sensitivity to thyroid hormone | CHEMBL2035874 | EPROTIROME | 3 | 7.20 |
| HP:0002930 | Impaired sensitivity to thyroid hormone | CHEMBL549748 | TETRAC | 0 | 6.72 |
| HP:0002930 | Impaired sensitivity to thyroid hormone | CHEMBL41632 | TIRATRICOL | 2 | 6.72 |
| HP:0002930 | Impaired sensitivity to thyroid hormone | CHEMBL1624 | LEVOTHYROXINE | 4 | 6.42 |
| HP:0002930 | Impaired sensitivity to thyroid hormone | CHEMBL1544 | LIOTHYRONINE | 4 | 6.42 |
| HP:0002069 | Bilateral tonic-clonic seizure | CHEMBL3809595 | NA | 0 | 6.25 |
| HP:0100723 | Gastrointestinal stroma tumor | CHEMBL1642 | IMATINIB MESYLATE | 4 | 5.95 |
| HP:0004319 | Decreased circulating aldosterone level | CHEMBL934 | METYRAPONE | 4 | 5.75 |
| HP:0012166 | Skin-picking | CHEMBL41 | FLUOXETINE | 4 | 5.55 |
| HP:0100697 | Neurofibrosarcoma | CHEMBL507361 | MIRDAMETINIB | 2 | 5.42 |
| HP:0005506 | Chronic myelogenous leukemia | CHEMBL1289926 | AXITINIB | 4 | 5.31 |
| HP:0002069 | Bilateral tonic-clonic seizure | CHEMBL507974 | TETRODOTOXIN | 3 | 5.28 |
| HP:0004319 | Decreased circulating aldosterone level | CHEMBL9298 | FADROZOLE | 0 | 5.28 |
| HP:0000079 | Abnormality of the urinary system | CHEMBL1560 | CAPTOPRIL | 4 | 5.28 |
| HP:0000726 | Dementia | CHEMBL116955 | CONGO RED | 0 | 5.18 |
| HP:0005506 | Chronic myelogenous leukemia | CHEMBL206834 | BAFETINIB | 2 | 5.18 |
| HP:0100723 | Gastrointestinal stroma tumor | CHEMBL276711 | SEMAXANIB | 3 | 5.10 |
| HP:0030078 | Lung adenocarcinoma | CHEMBL2347958 | NA | 0 | 5.07 |
| HP:0003076 | Glycosuria | CHEMBL3039507 | SOTAGLIFLOZIN | 4 | 5.02 |
