# Supplementary figures and images for: Better understanding the phenotypic effects of drugs through shared targets in genetic disease networks
Source: Front Pharmacol. 2025 Jan 22;15:1470931. doi: 10.3389/fphar.2024.1470931 (PMC11794328; doi:10.3389/fphar.2024.1470931)

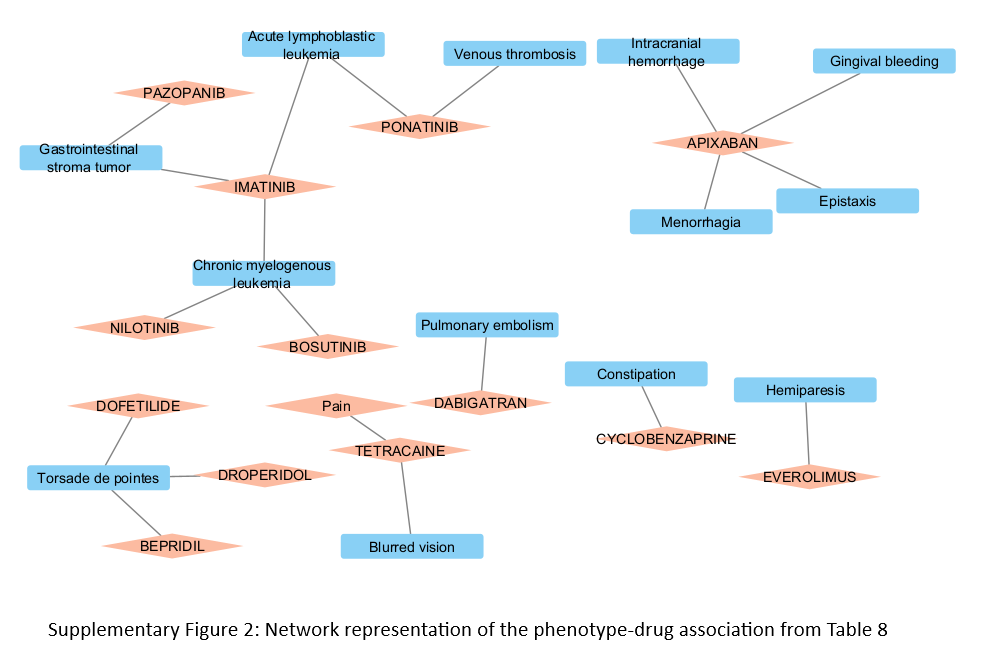

Supplement: Supplementary file 8 [file Image2.png]

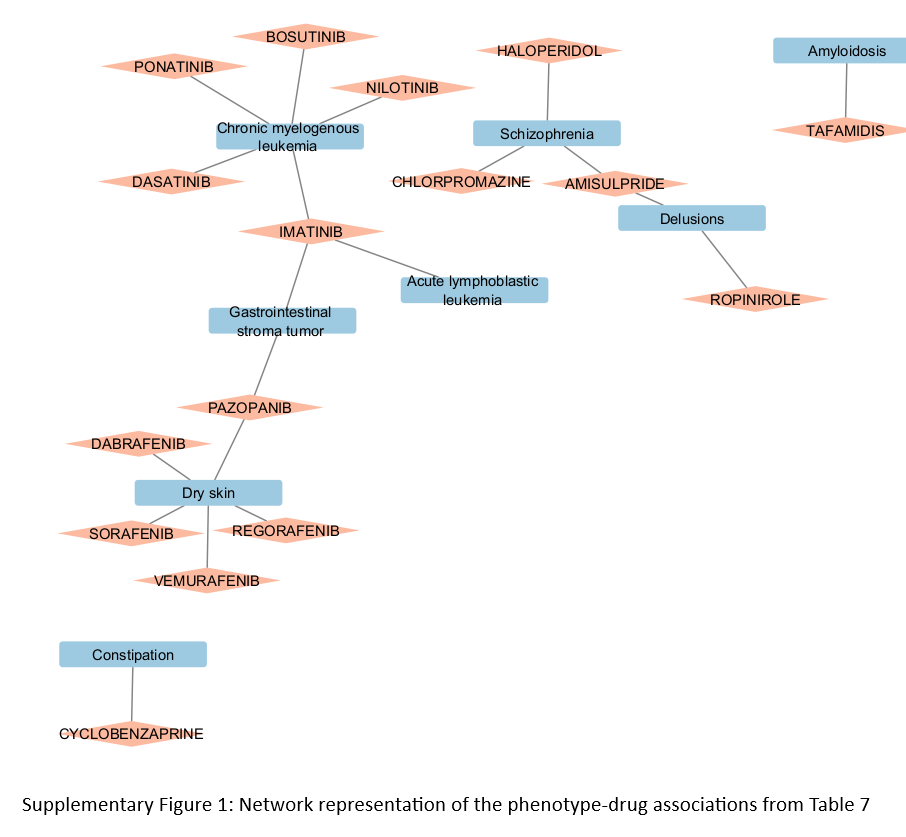

Supplement: Supplementary file 9 [file Image1.png]
