# Supplementary material for: Better understanding the phenotypic effects of drugs through shared targets in genetic disease networks
Source: Front Pharmacol. 2025 Jan 22;15:1470931. doi: 10.3389/fphar.2024.1470931 (PMC11794328; doi:10.3389/fphar.2024.1470931)
Supplement: Supplementary file 10 [file DataSheet8.pdf]

*Supp Table 8 Top drug-phenotype pairs according to the hypergeometric index, based on the Orphanet dataset using the domain-target based methodology, only including ChEMBL drugs with drug names found by literature comention Drug: ChEMBL database ID, HyI: hypergeometric index.*

| HPO | HPO name | Drug | Drug Name | Phase | HyI |
|---|---|---|---|---|---|
| HP:0001688 | Sinus bradycardia | CHEMBL45816 | MIBEFRADIL | 4 | 47.51 |
| HP:0001664 | Torsade de pointes | CHEMBL45816 | MIBEFRADIL | 4 | 47.03 |
| HP:0003712 | Skeletal muscle hypertrophy | CHEMBL4208190 | NA | 0 | 31.90 |
| HP:0001276 | Hypertonia | CHEMBL698 | TETRACAINE | 4 | 31.90 |
| HP:0003712 | Skeletal muscle hypertrophy | CHEMBL698 | TETRACAINE | 4 | 30.86 |
| HP:0007215 | Periodic hyperkalemic paralysis | CHEMBL4208190 | NA | 0 | 30.49 |
| HP:0031475 | Status epilepticus without prominent motor symptoms | CHEMBL4208190 | NA | 0 | 26.89 |
| HP:0003712 | Skeletal muscle hypertrophy | CHEMBL507974 | TETRODOTOXIN | 3 | 26.64 |
| HP:0003712 | Skeletal muscle hypertrophy | CHEMBL501134 | SAXITOXIN | 0 | 26.64 |
| HP:0012726 | Episodic hypokalemia | CHEMBL4208190 | NA | 0 | 26.22 |
| HP:0002121 | Generalized non-motor (absence) seizure | CHEMBL4208190 | NA | 0 | 25.83 |
| HP:0002121 | Generalized non-motor (absence) seizure | CHEMBL3809595 | NA | 0 | 25.83 |
| HP:0007215 | Periodic hyperkalemic paralysis | CHEMBL507974 | TETRODOTOXIN | 3 | 25.22 |
| HP:0007359 | Focal-onset seizure | CHEMBL4208190 | NA | 0 | 24.83 |
| HP:0007359 | Focal-onset seizure | CHEMBL3809595 | NA | 0 | 24.83 |
| HP:0002069 | Bilateral tonic-clonic seizure | CHEMBL4208190 | NA | 0 | 24.68 |
| HP:0002069 | Bilateral tonic-clonic seizure | CHEMBL3809595 | NA | 0 | 24.68 |
| HP:0025101 | Dysgenesis of the hippocampus | CHEMBL4208190 | NA | 0 | 23.90 |
| HP:0008151 | Prolonged prothrombin time | CHEMBL512351 | BETRIXABAN | 4 | 23.44 |
| HP:0008151 | Prolonged prothrombin time | CHEMBL206335 | RAZAXABAN | 0 | 23.44 |
